# Rhizoslides: paper-based growth system for non-destructive, high throughput phenotyping of root development by means of image analysis

**DOI:** 10.1186/1746-4811-10-13

**Published:** 2014-05-27

**Authors:** Chantal Le Marié, Norbert Kirchgessner, Daniela Marschall, Achim Walter, Andreas Hund

**Affiliations:** 1 Institute of Agricultural Sciences, ETH Zurich, Universitätstrasse 2, 8092 Zurich, Switzerland

**Keywords:** Root system architecture, Maize, High-throughput, Imaging, Rhizoslide, Breeding, Agriculture

## Abstract

**Background:**

A quantitative characterization of root system architecture is currently being attempted for various reasons. Non-destructive, rapid analyses of root system architecture are difficult to perform due to the hidden nature of the root. Hence, improved methods to measure root architecture are necessary to support knowledge-based plant breeding and to analyse root growth responses to environmental changes. Here, we report on the development of a novel method to reveal growth and architecture of maize root systems.

**Results:**

The method is based on the cultivation of different root types within several layers of two-dimensional, large (50 × 60 cm) plates (rhizoslides). A central plexiglass screen stabilizes the system and is covered on both sides with germination paper providing water and nutrients for the developing root, followed by a transparent cover foil to prevent the roots from falling dry and to stabilize the system. The embryonic roots grow hidden between a Plexiglas surface and paper, whereas crown roots grow visible between paper and the transparent cover. Long cultivation with good image quality up to 20 days (four fully developed leaves) was enhanced by suppressing fungi with a fungicide. Based on hyperspectral microscopy imaging, the quality of different germination papers was tested and three provided sufficient contrast to distinguish between roots and background (segmentation). Illumination, image acquisition and segmentation were optimised to facilitate efficient root image analysis. Several software packages were evaluated with regard to their precision and the time investment needed to measure root system architecture. The software 'Smart Root’ allowed precise evaluation of root development but needed substantial user interference. 'GiaRoots’ provided the best segmentation method for batch processing in combination with a good analysis of global root characteristics but overestimated root length due to thinning artefacts. 'WhinRhizo’ offered the most rapid and precise evaluation of root lengths in diameter classes, but had weaknesses with respect to image segmentation and analysis of root system architecture.

**Conclusion:**

A new technique has been established for non-destructive root growth studies and quantification of architectural traits beyond seedlings stages. However, automation of the scanning process and appropriate software remains the bottleneck for high throughput analysis.

## Background

The direct selection of efficient root systems is an important aim for a second green revolution enabling to increase yield in low input agriculture [1]. As costs of fertilizers rise and some fertilizers, especially phosphorus, become limited, there is an increasing interest in understanding the genetic control of RSA traits. Still, the ability to phenotype roots with sufficient throughput remains the bottleneck. Throughput is needed to close the phenotype-to-genotype gap, either by classical mapping of quantitative trait loci (QTL) or by association mapping [2]. In practice at least 100 – 500 individuals are needed for a QTL or association study [3]. Here we focus on the root system of cereal roots, especially maize. The root systems of cereals consist of three different below ground root types, the embryonic primary and seminal roots and the shoot borne crown roots [4,5]. The crown roots are separated from the embryonic roots by the mesocotyl, which elongates to place the shoot base close to the soil surface. While the embryonic root system can be studied directly after germination, crown roots in the example of maize develop around the two leaf stage [6].

Various phenotyping platforms were developed to monitor root growth non-invasively in soil and with high throughput in hydroponics, aeroponics, agar and on germination paper [7-10]. However, these platforms are limited to the assessment of the embryonic root system, disregarding, that the crown roots dominate the root system of a mature plant [11]. There is a certain risk that seedling root traits are of little relevance for the development beyond the seedling stage. For maize, there is strong evidence that the embryonic roots, especially the primary root, behave differently compared to the crown roots. All three root types (primary, seminal and crown roots) are under different genetic control [12] and the early development of embryonic roots is affected strongly by seed size, seed quality, timing of germination and other processes as discussed by Hund et al. [5].

There is a lack of high throughput phenotyping methodologies enabling to study crown roots and their response to environmental stimuli. The reason for this is that cultivating plants with accessible crown root systems is far from being trivial: Containers need to be larger, require more space, and the access and measurement of the root systems requires more time. Different approaches were taken to access root systems of larger plants. Soil based systems such as rhizotrons and containers [13,14] or systems observing roots in undisturbed soil in pots (e.g. via computed tomography; [15] or magnetic resonance imaging; [16]). Usually these systems offer high precision but have limited throughput or require major investments for automation as in the case of GROWSCREEN Rhizo [17]. Otherwise, soil free systems are preferred for large-scale genetic screens, as they can be assessed more rapidly and enable for a sufficient number of replicates [7,9,18-21]. One widely used soil free cultivation method is to grow plants on germination paper. Such paper is not only used for routine germination testing but also to assess root traits as it is easy to handle, can be kept free from pathogens and enables to manage a high number of replicates on a limited space. Moreover, the access to the root system is simple and coloured paper, unlike soil, provides favourable optical contrast between background and roots, thereby making automatic digital image processing possible [10]. On germination paper a wide range of experiments were performed. Investigations focused e.g. on the interaction between roots and rhizobacteria [22] as well as on the effects of temperature [23], low water potential induced by polyethylene glycol [24,25], nutrient deficiencies [26,27] and aluminium toxicity [28] on root growth.

The paper-based systems developed so far have the above mentioned disadvantage, that only the early embryonic root system can be assessed. Several factors complicate the enlargement of such systems in order to assess the development of crown roots. The most critical point is the increasing overlap and parallel growth of roots on the two dimensional paper surface. To circumvent this, different root types may be grown in a layered sandwich of paper as we will outline in Methods. Here we describe the biological basis of this approach: The mesocotyl, situated between the scutellar node bearing seminal roots and the first node bearing crown roots, elongates to place the shoot base at the soil surface. Mesocotyl elongation is stimulated by darkness [29]. If seeds are placed in the dark between two papers, the embryonic roots are growing between the papers, while the mesocotyl elongates and places the crown roots on top of the papers.

For a sufficient throughput, not only the cultivation method, but also image acquisition and image analysis are of high importance. A sophisticated image analysis is essential for fast and meaningful RSA analysis [30]. The process of image acquisition needs to be optimized with respect to an optimal contrast between roots and background and with respect to a sufficient spatial resolution to clearly visualize and quantify also finer roots with small diameter. Past studies showed that hyperspectral data can be used to elucidate differences between soil and roots or to identify plants infected with root rot [31,32]. In this context, it is important to identify wavelengths with an optimal contrast between root and background.

A wide range of literature is available dealing with software that enables for image based root system analysis [33-45] and there is an online database comparing the different software packages that are already available [46]. Yet, in the context of our study, it is crucial to explain how the optimal software should be chosen for the purpose of parameter extraction on rhizoslides, to clarify under which circumstances such a software performs best and to outline putative pitfalls. Available software for RSA analysing ranges from completely automated analysis delivering global root data via semi-automated systems to hand measurements enabling detailed measurement of a wide range of traits [33,34,36-40,42-45,47].

In summary, growth pouches as described by Hund et al. [10] have the disadvantages that i) only the early, embryonic root system may be studied and ii) an intense user interference is required to mount the pouches on the imaging station and open opaque foil covering the roots. The aim of this project was to develop a paper-based root observation system, so called rhizoslides, that enable for i) a characterization of post-embryonic cereal root systems and ii) automated or semi-automated image acquisition and processing.

## Results

### Root slides enable the separation of crown roots

**Figure 1:**
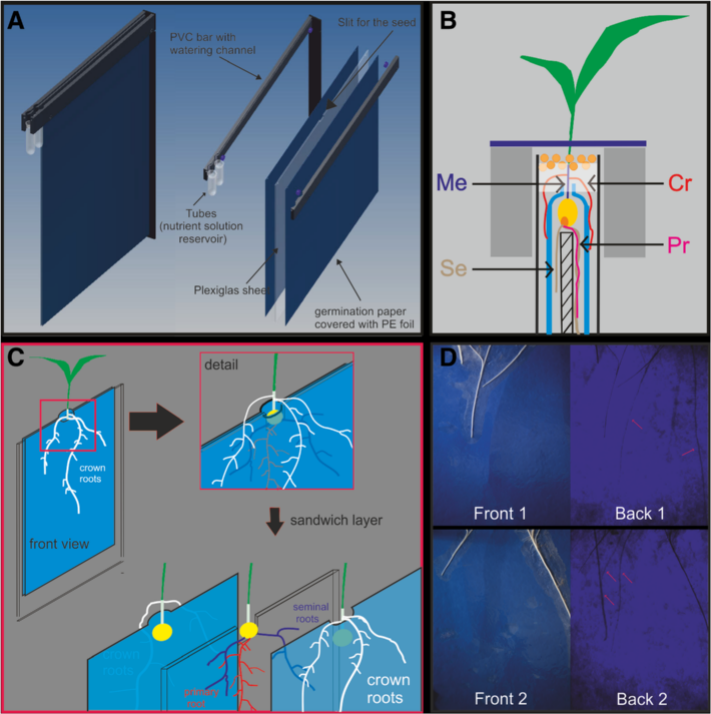
**Construction of the rhizoslides. A**: Root slides consistent of a plexiglass sheet covered with germination paper and a transparent PE foil belt with PVC bars with watering channels. Tubes on the site serve as nutrient solution reservoir. **B**: Cross section of the rhizoslide, illustrating the seed placement and separation of embryonic and crown roots. The seed is placed between the germination paper and the space between the PVC bars is filled with a layer of Potassium polycarbonate and a granulate substrate. Me: Mesocotyl; Cr: Crown root; Se: Seminal root; Pr: Primary root. **C**: Schematic figure of the separation of the embryonic and shoot-born crown roots: Embryonic roots are growing hidden below the germination paper whereas crown roots are growing visible on the top of the germination paper. **D**: Images taken of one slide with front- or backlight. Front 1 and Back 1 (complementary Front 2 and Back 2) are images of the same side taken with either front or backlight. Front images show crown roots whereas the transmitted light allows detection of seminal roots as well.

The root slides enabled to grow maize seedlings to three fully developed leaves before the first embryonic root reached the bottom edge of the paper. The nodal root system was observed until the four-leaf stage. The plants needed 10 and 20 days to reach the three and four leaf stage, respectively. A separation between embryonic and postembryonic roots was achieved by physically separating roots into the different layers of the rhizoslide sandwich construction. Embryonic roots were growing in the invisible layer between the plexiglass sheet and the germination paper (Figures 1A, B and C), whereas the crown roots grew in the outermost, visible layer, on top of the germination paper covered with a transparent PE foil (Figure 1C). 90% (= 41 roots of 9 plants) of the crown roots grew on top of the germination paper and only 10% (=4 roots of 9 plants) between the paper and the plexiglass (See Additional file 1). The embryonic roots grown under the germination paper could be visualized using backlighting (Figure 1D). Tested alternatives to the separation of embryonic and postembryonic roots were to grow them not separated but either on both sides of the plexiglass sheet on the germination paper or on one site of the plexiglass sheet. The advantage of this method is the opportunity to monitor all root types at the same time without the usage of backlighting. The disadvantage is that space is very limited and roots start to grow parallel and cross each other. With increasing age, root tracing became demanding. Furthermore, fungal growth was successfully suppressed by moistening the germination paper with the fungicide Captan and by adding Captan to the nutrient solution. The nine replications of the control slides (no fungicide) were all colonised by fungi whereas both Captan concentrations (2.5 g L^-1^ and 5 g L^-1^) reduced the colonization to one out of nine slides (See Additional file 2). Most fungi on the non-treated paper were *Chromelosporium fulvum* (See Additional file 3). Total root length did not differ between the treated and non-treated plants (data not shown), but plant development was delayed compared to the control plants (See Additional file 2).

### Reflections are overcome using polarization filters and a staggered flash

**Figure 2:**
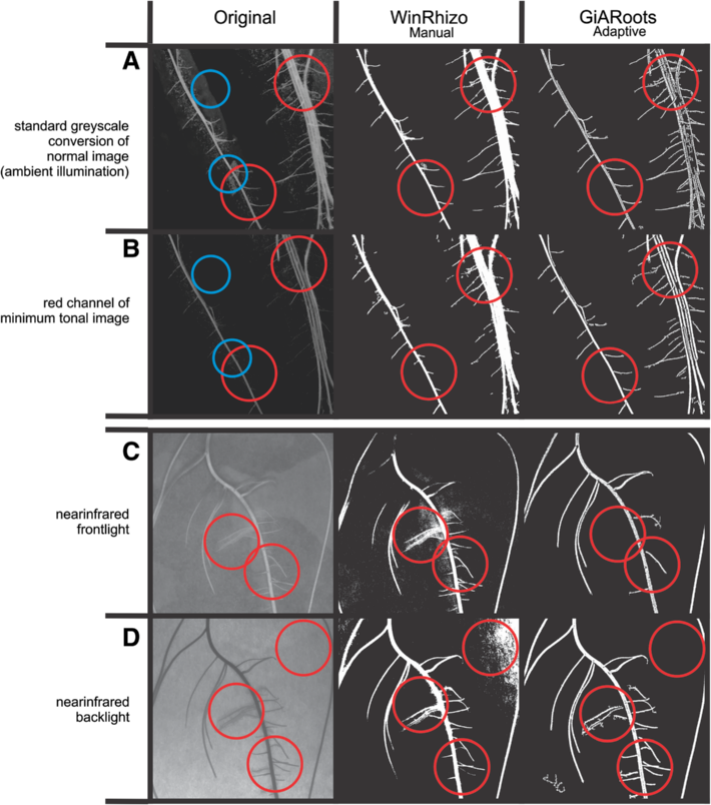
**Imaging and thresholding methods.** Images of roots grown on either Anchor blue (A + B) or Sebio grey (C + D). Red circles highlight regions for which the different thresholding methods yielded contrasting results (Lateral roots vanished, parallel growing roots became one root or the intensity of background noise). Blue circles indicate the removal of droplets and reflections. **A**: Image taken with diffuse lighting. **B**: Two images taken with flash light (right/left side) including polarization filters on flash and camera lens and combined to a minimum tonal image. The red channel was used for the conversion into greyscale. **C**: Image taken with near-infrared front lighting. **D**: Image taken with near-infrared backlighting. Thresholding was done using the WinRhizo or GiARoots routines. Only the routines resulting in the best separation between root and background are shown.

We aimed to optimize image acquisition to enable imaging through the transparent cover foil with a minimal disturbance or reflectance of light, haze or droplets on the surface of the foil.The minimum tonal value method, i.e. combining the left and right image by keeping only the minimum tonal value present in either image, resulted in a lower amount of reflections of the bends in the surface of the covering transparent foil (Figures 2A and B; upper blue circle) and a reduction of reflections by droplets (Figures 2A and B; lower blue circle). It also increased the contrast between roots and background compared to ambient illumination (Figures 2A and B). The higher contrast presumably resulted from the shadows from left and right illumination, which were retained in the combined image. A further advantage of the shadows was a better distinction between roots growing in parallel (Figures 2A and B; upper right red circle). A slight disadvantage was that the detection of the origin of lateral roots became more difficult as they emerged in the shadowed region (Figures 2A and B; lower left red circle).

### Red light created the strongest contrast

**Figure 3:**
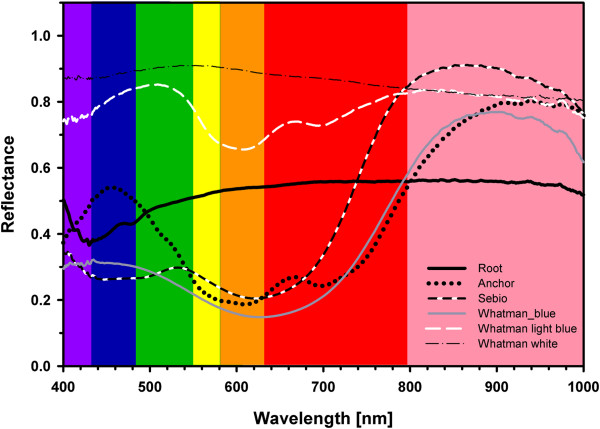
**Hyperspectral reflectance of root and paper.** Reflectance of the root and five tested germination papers (Anchor, Whatman blue, light blue, and white and Sebio grey) in the spectrum from 400–1000 nm. Colouring indicates the spectral range of blue, green and red light. Slightly red coloured is the near-infrared range (790–1000 nm).

We used spectral reflectance to elucidate at which wavelengths the contrast between roots and paper background is maximized. Based on this information we aimed to identify which colour channel of the available camera would be best suited to segment between roots and paper background. The reflection of germination paper behaved differently depending on colour and/or texture and there were differences in reflectance between the root and the papers (Figure 3). The root reflected in the entire range between 400 and 1000 nm with small differences in reflection intensity. A similar pattern was observed for the white Whatman paper, where the intensity was two times higher than for the root. Also the light blue Whatman paper showed a higher reflection compared to the root and reflection maxima were situated in the blue and infrared range. All strongly blue and grey colored papers (steel blue (Anchor), Whatman blue, Sebio grey) showed a similar pattern of a high reflection in the blue range, a decrease in green and red and an increase in the near-infrared range. With the exception of the steel blue paper, the intense reflection of these papers in the blue range was lower than the reflection of the root. Between 560 and 720 nm the reflection of all three blue papers was two times lower compared to the reflection of the root (Figure 3). Depending on these results, images were taken and colour channels tested. The best distinction between root and background was obtained with the red channel for steel blue, Whatman blue and Sebio grey (See Additional file 4A). Whatman white showed similar poor results for all three channels.

### Near infrared backlight enables root growth studies

Images taken in the near-infrared range (940 nm) confirm the observation of a slight contrast between root and paper due to a high reflectance in the near-infrared range of the papers. Best results were obtained using Sebio grey paper (see Additional file 4B), but the low contrast and noise due to reflections resulted in a loss of lateral roots (Figure 2C). However, using near-infrared back lighting, segmentation between root and background was good and only negligible losses of root structures occurred (Figure 2D). However, both thresholding methods still had problems to handle the background noise resulting from the paper texture. Compared to other papers, the Sebio paper had the advantage of a fine texture and thinness, which produced only slight background noise in the backlight image (Figure 2D). In contrast, the texture noise of the steel blue germination paper (Figure 1D) was too high for segmentation methods applicable to date.

### Image analysis software

We conducted a literature search to identify software with the ability to perform a RSA analysis of complex root systems grown on two dimensional images. As a result, eight potentially suitable software packages were chosen (See Additional file 5) and three of them could be successfully installed and tested (all software was downloaded in October 2012). Two of them, WinRhizo (WinRhizo Pro 2009b, Régent Instruments Inc.) and GiARoots [37] offer batch processing with the opportunity to perform manual adjustments. The third software, SmartRoot [40] is semi-automated. We were not able to test the remaining five software packages for different reasons: DigiRoot [48] and RootReader2D [7] could be successful installed, but did not accurate work with the supplied material (incomplete/wrong marking of the roots). EZ-Rhizo [33] could be successful installed, but the software stopped working immediately after starting the analysis. We tested whether it would work with different image formats or resolution and requests assistance from the developer, which remained unanswered. RootTrace [42] could not be successful installed in spite of intense support by the developer. The software DART [39] could be installed, but not opened. Our help request was answered with the comment that the software was not developed to a stage where it would stably run on any system.

### GiARoots facilitates best automatic thresholding routine

**Figure 4:**
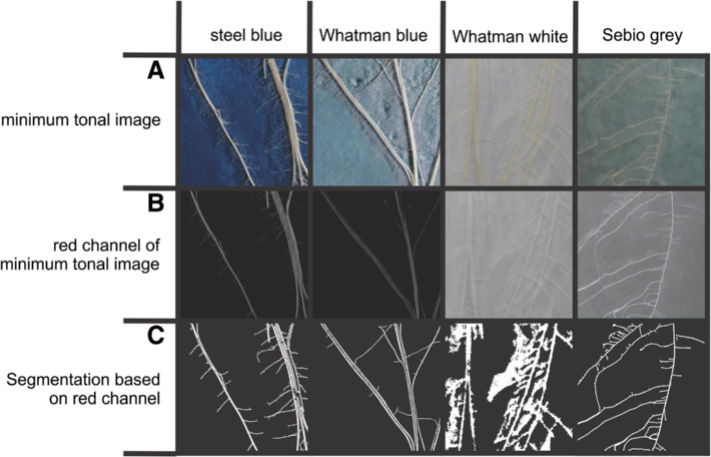
**Application of optimal image processing.** The optimized image processing protocol as described in Figure 6, was applied to the four most promising papers identified based on spectral imaging (Figure 3). **A**: Images of roots grown on Anchor, Whatman blue and white or Sebio paper. Two images were taken with flash light from the right/left side and combined to one image. **B**: Conversion into greyscale using the red channel for conversion. **C**: Segmentation of the root system using the adaptive threshold of GiARoots.

In addition to improve image quality, we evaluated different methods for image segmentation. The thresholding methods of WinRhizo and GiARoots were tested and compared. These two software packages were chosen because they make an automated conversion into binary images possible and worked well with the supplied material. SmartRoot was not included in this part of the analysis, as it works on the basis of greyscale images. In WinRhizo, the automatic routine did not eliminate all background noise; in contrast the manual adjustment facilitated a better reduction of the background noise without a loss of information (See Additional file 6). In WinRhizo, it was not possible to distinguish roots growing in parallel to each other (See Additional file 6, blue circles). However, the software GiARoots enabled to some extent distinguishing between roots growing in parallel, but the images of the segmented roots were incomplete, making it difficult to identify individual roots (See Additional file 6, blue circles). Both routines (adaptive and double-adaptive threshold) enabled a good separation between root and background and no big difference was observed between them (See Additional file 6). Lateral roots with a weaker contrast were clearly visible after adaptive thresholding in GiARoots and manual adjustment of the threshold in WinRhizo, but they were neither detectable using the global threshold value in WinRhizo, nor the double adaptive threshold in GiARoots (See Additional file 6; red circles). Based on these results, images taken on either steel blue germination paper, Whatman blue, white or Sebio grey were transferred into greyscale images using the red channel and were segmented using the adaptive threshold of GiARoots (Figure 4). The best results, regarding the ratio (reduction of background noise)/(loss of lateral roots), were obtained on steel blue germination paper and Sebio grey. For both papers a good separation between root and background was obtained with a minor loss of lateral roots.

### High repeatability of SmartRoot

To determine the influence of the user on the results using semi-automated software, a test for repeatability was done using SmartRoot.

In SmartRoot, the variance of root length detected in ten different images was related to the overall variance created by different users and the interaction between users and image content. Image processing was highly repeatable with respect to the overall length detected for the lateral roots (repeatability of 0.99) and axile roots (repeatability of 0.97). Total measured length of lateral roots ranged from 152 to 164 cm; total length of axile roots varied from 162 to 165 cm, depending on the user.

### Good correlations for SmartRoot and WinRhizo

**Figure 5:**
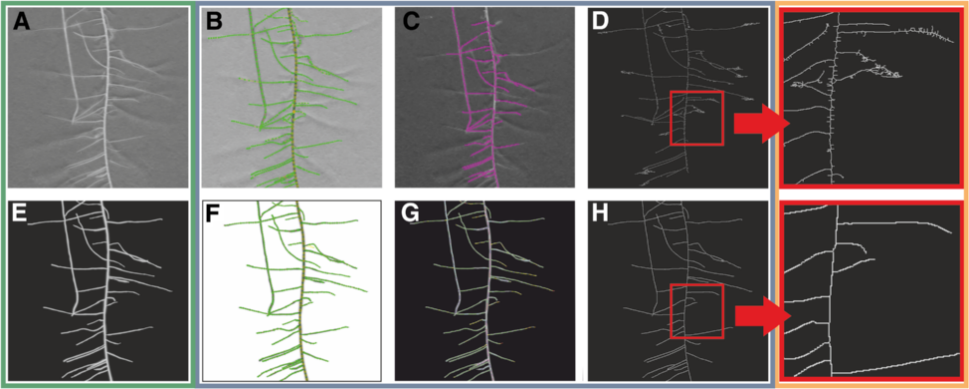
**Tracking of the root system by the three software packages. A**: Greyscale image of a root system section. **B-D**: Tracking of the roots in SmartRoot **(B)**, WinRhizo **(C)** or GiARoots **(C)** using the image shown in **A**. **E**: Artificial root of **A** derived from vectorization in SmartRoot. **F-H**: Tracking of the roots in SmartRoot **(F)**, WinRhizo **(G)** or GiARoots **(H)** using the binary image shown in **E**.

As SmartRoot enables for a user-defined, controlled tracing of the whole root system we considered the output of this software package as the one that represents best the real root system length. Compared to SmartRoot, WinRhizo underestimated the total root length due to the fact that it could not detect lateral roots that only showed a small contrast between root and background (Figures 5B and C). In contrast to this, GiARoots rendered much higher root lengths. Hereupon, we evaluated the images showing the thinned objects and observed thinning artefacts (Figure 5D). We anticipated that these differences were a result of artefacts caused by root hairs, reflections and other effects leading to a ragged edge between root object and background. Therefore, the output would be comparable under optimal image conditions, defined by smooth edges of the roots and by a perfect contrast between root and background. To test this hypothesis, we reconstructed the root systems traced in the ten images using the data of the vectorized root system supplied by SmartRoot. This resulted in ten images of “artificial roots” with an optimal contrast and a known length. Indeed, visually all programs detected the roots without losses or false tracing (Figure 5E-H). However, there were still differences in total root length. The values obtained with SmartRoot differed 0–5% compared to the original pixel length, WinRhizo differed 0–4% and GiARoots differed 2–22%. Even more important than the absolute values are the correlations between the results obtained with the three programs. For the artificial root images, the correlations between all programs were satisfying with r^2^ values between 0.91 and 0.97 (See Additional file 7B), but for the original images, the correlations were much lower (0.33 GiARoots-WinRhizo; 0.54 WinRhizo-SmartRoot; 0.67 GiARoots-SmartRoot) (See Additional file 7A). In addition to total root length measurements, all three programs enable to study further traits of root system architecture. As the measured traits and methods differed strongly among the software packages, they could not be used for software comparison. Total root length was chosen as a common trait for differentiating the performance of the software packages. Other traits such as lateral root number, angle between roots etc. were less good indicators of the performance of a software package. As already shown, software packages often underestimate the length of a certain lateral root; hence, their applicability increases with increasing length of each lateral root, and, therefore with total root length. Similar examples are traits based on diameter calculations as surface area (cm^2^) or volume (cm^3^). Correlations for the root diameter were low for all three programs (between 0.025 and 0.51) for the original images and consequently for calculated traits based on the diameter as well (See Additional file 7A). Diameters measured by SmartRoot and WinRhizo correlated for the artificial roots (0.96), but for GiARoots the correlations remained low with both programs (0.07; 0.08) (See Additional file 7B). As WinRhizo also enables for topology analysis, the time investment for a topology analysis in WinRhizo as well as for SmartRoot was investigated. The images were taken from root systems of plants with two fully developed leaves grown in small pouches (21 × 29.5 cm). These data set has been described previously [10]. The analysis was divided into four steps and time investment for each step was recorded. For both programs the last step (lateral root tracing/assignment of ranks) was most time consuming (See Additional file 8). The analysis of the images used in our study took between 8 and 40 minutes depending strongly on the number of lateral roots. A correlation between number of lateral roots and the required time for analysis could be observed for WinRhizo (R^2^ = 0.76) and SmartRoot (R^2^ = 0.44). Although both programs enable topology analysis, the obtained traits are not identical and not all traits are immediately extractable from the output but must be calculated (See Additional file 9). An advantage of SmartRoot compared to WinRhizo is the clear arrangement of the output file. This facilitates to obtain information for every single root immediately (See Additional file 10B). In contrast, in the WinRhizo output every measured section (link) is listed and the data needed to be summarized by the user first, before meaningful traits can be extracted (See Additional file 10A). This data reorganization is very time intensive. GiARoots does not enable for a manual topology analysis as all images are batch processed.

## Discussion

The aim of this work was to create a growth system that enables for non-destructive and potentially high-throughput quantification of root system architecture traits. Ultimately, this system should be applicable for genome mapping of crown root characteristics. The “sandwich” composition of the paper layers enabled distinguishing between embryonic and postembryonic roots as they grew in different layers. Postembryonic crown roots are a major focus, as they account for the major part of the adult root system [11], and the ability to study their response to stresses offers a major advantage.

The rhizoslide sandwich system is a good compromise between the space-saving and handling capacities of a 2D-system and the advantage of 3D-systems enabling for an unlimited spread of roots in three dimensions. Single layered systems, even if up scaled to larger paper size, have the disadvantage that roots will increasingly overlap and crown roots will be difficult to measure. Three dimensional systems based on agar, aero- or hydroponics circumvent these problems [7-9]. Agar has got the advantage that roots stay in place and do not overlap. However, space is usually limited and keeping the agar free of pathogens is laborious. Therefore, studies are preferably performed over a short time period. Hydro- and aeroponic cultivation make it possible to studying a high number of individuals over a long time period, but roots change their position and this complicates the image analysis.

A big advantage of the rhizoslides is that they facilitate studying the response of particular root types, especially crown roots, to changes of the root environment in space and time. For example, different concentrations of nutrients can be applied to the paper on the left and right side of each slide. In split-root setups responses to stimuli such as altered nutrient availability can be studied [49-51]. Besides root system architecture traits, the plasticity and the dynamic alteration of root growth to changing environments can be observed. Previous studies showed that root morphology and growth can change in acclimation processes to nutrient availability as short as well as long term response [52-55]. Rhizoslides offer the potential to study such response on a large number of plants. In the past, studies were done on monocot species [10,56-58] as well as on dicot species [59] using the so called paper-roll setup or growth pouches. For example, Watt et al. grew wheat seedlings in a paper-roll setup and found positive correlations of root length between seedlings grown in the paper-roll setup or in the field, but not with the reproductive stage [56]. These findings emphasize the importance to work with later developmental stages. Potentially the paper-sandwich is perfectly suited to study the fibrous root system of monocot species e.g. rice, wheat, or barley. The mesocotyl elongation is used to separate embryonic from crown roots. Eventually, small adjustments (e.g. a smaller slit in the plexiglass sheet) have to be made to keep the smaller seeds in space and to ensure an elongation of the mesocotyl above the paper edge. For dicot species, which form one tap root undergoing secondary thickening, the sandwich system is less suited. Still such roots can be studied on one site of the Plexiglass sheet. This has the already mentioned disadvantage that space is very limited and roots start to grow parallel and cross each other. Furthermore, in the current rhizoslide version, the Plexiglass plate bends, as the adhesive power of the nutrient solution connects it tightly with the paper and the covering foil. Apparently, each material has a different coefficient of expansion. Therefore, it is advantageous to grow either two plants on one plate (each on one side) or to enable root growth on both sides of the plate.

Furthermore, the adjustment of parameters of the rhizosphere, such as pH, CO_2_ or O_2_ can be analysed via Optodes in complementation to growth analysis [60]. Rhizoslides are not only an opportunity to perform high throughput screening for RSA traits, as usually done for QTL mapping, but allow to do more precise effect studies on a small scale with high temporal resolution. The lack of automation is currently the only bottleneck to achieve high temporal resolution.

We optimized the imaging system to enable automation. The necessity to remove the foil covering the roots in order to avoid reflections of the cover itself and of droplets on the inside of the cover was a major bottleneck hampering automation. These reflections could be successfully minimized by using polarization filters in combination with the combined images with left and right illumination, respectively. Polarization filters are commonly used to reduce noise due to reflections and were already successfully used by Clark et al. [7] in a hydroponic system. We took this approach further, by combining two images, each illuminated from a different angle. By retaining only the lower (darker) pixel value of each image in the final minimum tonal image, we utilized the optical properties of the three dimensional structures in the image: bright reflections on round droplets or on folds in the covering foil appeared at different locations depending on the illumination. These artefacts were minimized. Similarly, the cast shadows of the roots appeared either on the left or the right side and were maximized. Accordingly, the minimum tonal image had a strong local contrast between roots and background combined with reduced noise due to reflections. The possibility to take images without removal of a cover is a major advantage compared to the pouch system described by Hund et al. [10] which had to be opened manually. However, background noise by condensed water could not be completely removed by image combination, but by usage of backlighting.

Maximal contrast between roots and paper background can be achieved by using the red channel of the RGB images. This conclusion is based on our analysis of spectra of the root compared to those of various paper backgrounds. Hund et al. (2009) reported the saturation channel to be best suited for image segmentation. However, differences between the red and the saturation channel in the earlier study were very small. Several studies showed that illumination within the visible range affects root growth [29,61,62]. A frequently used alternative is near-infrared (NIR) light as so far no negative effect on root morphology could be observed [63,64]. NIR illumination was used to differentiate between roots and soil background [32] and to illuminate roots grown in aeroponics (personal communication, Draye, X.). In Rhizoslides only NIR backlighting resulted in a sufficient contrast and, with a double layer of thick steel blue germination paper, this contrast was not sufficient for segmentation methods available to date. This makes NIR unsuitable as light source in paper-based rhizoslides. Furthermore, in previous studies with growth pouches, the influence of the scanning light on root growth during imaging was negligible (Hund et. al 2009). It remains to be tested, whether an increased frequency of illumination in high-throughput screening approaches would have systematic effects on root morphology.

The resolution of the camera was high enough to detect first-order lateral roots of maize. Given the dimension of the imaged area of the slide of 490 mm widths in combination with the 21 mega pixel camera, a pixel size of 0.13 mm was achieved. A minimum of three pixel are required to detect roots by means of an image processing software. This three-pixel diameter of 0.39 mm is in the range of the lateral root diameter of maize. Hund et al. (2004) reported lateral root diameters of maize in the range of 0.26 to 0.47 mm in plants grown in sand substrate under chilling conditions. The diameters of lateral roots in pouches are usually below a threshold value of around 0.5 mm [10,24] with average diameters ranging between 0.25 and 0.306 mm [23]. However, Mac Cully et al. (1987) reported lateral roots as thin as 0.07 mm which would be below the threshold detected by the current setup. Accordingly, it will not be possible to distinguish between lateral roots diameters and to detect very fine root. The solution would be higher resolution. The four times smaller A4-size growth pouches in combination with a 28 megapixel scanner [10], yield an almost tenfold resolution of 0.042 mm px^-1^. By stitching multiple images or zooming into particular regions of interest, resolution on rhizoslides can be increased to a point where even monitoring of root hairs may be possible.

Suitable software remains a bottleneck. The three software packages, offered different strengths but had also severe weaknesses. Dependent on the research question WinRhizo and GiARoots offer the advantage of simple batch processing without additional user interference. The thresholding algorithm of GiARoots is more advantageous compared to WinRhizo when it comes to the elucidation of inhomogeneities in the root system. Accordingly, GiARoots provided a much better global segmentation. However, we did not test the color analysis in WinRhizo as an option for enhanced segmentation. After segmentation, GiARoots delivers basic characteristics of a root system with the lowest time investment and without influence of the user. A negative point for GiARoots is that it needs images with a good contrast to avoid false tracing. These artefacts may lead to a serious overestimation of total root length. The images derived from our rhizoslides did not provide sufficient contrast to avoid such artefacts. The automatic routines in WinRhizo provide root lengths that can be grouped in user-defined diameter classes. Using this root-length in diameter class distribution, roots may be classified in large-diameter axile roots and small diameter lateral roots [10,65]. This approach was efficiently used for high throughput image analysis in genome mapping studies [25,66,67] and it may be applicable for rhizoslides. WinRhizo and Smart Root offer the possibility to perform an in-depth topology analysis. For such an analysis an intense user interaction is needed to allocate lateral roots to their parental origin. For both WinRhizo and SmartRoot manual tagging of root for topology analysis is time intensive and ranges from 8 to 40 min for a root system grown on a small 21 × 29 cm paper. In the four times larger rhizoslides, a much higher time investment is needed unless the focus is on individual, representative roots. Furthermore, the user might bias the results as a high degree of user interaction is required. Although we could not detect strong bias among the three different test persons we recommend controlling potential systematic differences among users by an appropriate experimental design. A difficult part of the topology analysis in WinRhizo is the extraction of information from the generated output, as it is not intuitive and traits such as root length of single roots must be calculated. Furthermore, WinRhizo’s topology analysis does not allow for a simplified tracking of roots though multiple images of a time series. These difficulties could be the reason, why it was not used for genome mapping in crops to date and only in a small number of topology studies [68,69], as far as we know. SmartRoot offers both, a topology analysis with an intuitive output of the summary statistic and the possibility to tag and track roots through successive images of a time series [46]. The software was developed to enable more complex analysis of the RSA to do QTL analysis amongst others.

For many research questions, global root traits or quantitative traits as generated with automatic routines in WinRhizo or GiARoots are sufficient. A study with hundreds of individuals as needed in QTL analysis, for example, would require massive investment of time for manual root tracing. However, SmartRoot may prove suitable if only parts of the root system, e.g. some representative crown roots are to be measured in more detail. Such an approach would be feasible, even for quantitative genetic studies. For example, Trachsel et al. [66] measured the length of the primary axile root on more than 1000 plants using the ruler tool of Adobe Photoshop. As SmartRoot allows tagging and tracing of individual roots in image series in a convenient manner, it is particularly suited to monitor temporal changes in growth rates. Furthermore, there are research questions with a smaller number or repetitions that need to study e.g. single root scale. For these cases a program such as SmartRoot is optimal. To sum up, so far there is no optimal software solution for every setup. Instead the most suitable method must be chosen depending on the research question and the maintainable time investment.

## Conclusions

A new technique has been established for high-throughput non-destructive root growth studies and quantification of architectural traits beyond seedlings stages. The method allows studying root growth of crown roots and seminal roots independently under heterogeneous environmental conditions. Transparent foil sheets covering both sides of the sandwich construction allow for a rapid screening of the maize root system growing within the rhizoslide. In future, the usability for other crop species should be tested and necessary adaptations identified. The reflections of the foil could be successfully eliminated by a newly developed imaging setup and image processing. In future, rhizoslides can be used to study a wide range of research questions on a small scale as well as with a high number of replicates necessary e.g. for QTL analysis. A future challenge will be the establishment of a system allowing the automation of the imaging process to increase the screening speed of huge sets of genotypes. Of the tested software packages, each offered specific strengths. Specifically, we identified the segmentation algorithms of GiARoots to be optimal, we found the most precise automated measurement of root length using WhinRhizo and we saw a user friendly topology analysis combined with the ability to trace roots in successive images as the major advantages of SmartRoot. Improved next generation software solutions should ideally combine these strengths.

## Methods

### Plant material

All experiments were carried out with the maize hybrid Bonfire supplied by Delley seeds and plants Ltd (DSP Ltd), Switzerland.

### Materials

The rhizoslides (version 2.7) consist of two PVC bars (600 × 60 × 10 mm) and an acrylic sheet (530 × 650 × 4 mm) fixed with two screws between the bars (Figure 1A). Between acrylic sheet and bar, an 8 mm flat washer was placed to obtain a slit for the roots (see Additional file 11). On one side of the bars, 25 mL PE tubes (Semadeni AG, Ostermundigen, Switzerland) were placed to act as water/nutrient solution reservoirs (Figure 1A). On the inner side of each bar, a channel was mortised to hold a watering system. The watering system consisted of two glass fibre wicks (∅ = 2 mm) (Suter-Kunststoffe AG, Fraubrunnen, Switzerland), each surrounded with a PVC tube (outer diameter 5 mm; inner diameter 3 mm) (GVZ-Gossart AG, Otelfingen, Switzerland). The wick system allowed the transport of the nutrient solution via capillary force from the two reservoirs to the right and the left sides on the germination paper, respectively. The acrylic sheet was covered with wet germination paper (490 × 610 mm) on both sides serving as substrate. These were in turn covered by a transparent oriented polypropylene (OPP) foil with micro holes of 70 μm to allow for gas exchange (Maag, GmBH, Iserlohn, Germany). The foil is widely used in the packaging industry for cooled, fresh food to allow for gas exchange and to avoid droplets and fog on the transparent cover. Steel blue germination paper (Anchor Steel Blue Seed Germination Blotter, Anchor Papers Co, USA) (Anchor) proved useful in several studies evaluating root growth and development in growth pouches [10,20,23-25,28,70,71]. Unless mentioned otherwise, this paper was used for all standard tests. In addition, we tested four alternative germination papers with respect to their optical contrast to the root objects, i.e. light blue (FP3621), blue (FP3644), and white (FP5703) germination paper by Whatman (GE Healthcare Life Science, Glattbrugg, Switzerland) and Sebio grey (FP3236, Albet-Hahnemuehle S.L, Dassel, Germany).

### Cultivation conditions

For sterilization, the germination paper was heated in three cycles from room temperature to 80°C and kept at this temperature for at least 120 min. Between the heating periods the paper was kept for 20–22 h in an oven at 37°C and 50% relative humidity [72]. Maize seeds were surface sterilized with sodium hypochlorite for 15 min. and rinsed with deionized water for 5 min. Subsequently, seeds were kept for 48 h at 26°C in the dark for germination and were then transferred into the rhizoslides. To prevent fungi growth, a method described by Bohn et al. [73] was used. The germination paper was moistened with water containing 2.5 g L^-1^ Malvin (Syngenta Agro AG, Dielsdorf, Switzerland) containing the active component Captan. Plants were cultivated in a climate chamber (PGW36, Conviron, Winnipeg, MB, Canada) refurbished with new control unit, compressor and slight ceiling (Kälte 3000, Landquart, Switzerland). Environmental settings were a day period of 14 h light, at a temperature of 26/18°C (day/night) at seed level, 60% humidity and a light intensity of 230 μmol photosynthetically active radiation m^-2^ s^-1^ at plant canopy level supplied with a mixture of 2/3 Cool White (Philips TL5 HO 54 W/865; Philips, Zurich, Switzerland) and 1/3 GRO LUX (Sylvania F36W/GRO, Sylvania, Germany, Munich) light tubes.

### Root type separation into rhizoslide layers using mesocotyl elongation

The placement of the seed was done immediately after germination to avoid damaging the primary root. The seed was placed between the germination papers into a slit on the upper edge of the plexiglass sheet. The paper edge was placed at the plexiglass edge, surrounding the seed (Figure 1B). Paper clips or paper fastener stuck the two papers on the site of the seed. For mesocotyl elongation, the seed was kept in the dark. To keep the seed and mesocotyl wet Potassium polycarbonate moistened with deionized water containing 2.5 g L^-1^ Captan was injected between the PVC bars on top of the seed. To avoid incidence of light, a granulated substrate was placed on top of the Potassium polycarbonate and a cover was placed on top of the bars (See Additional file 12). To test whether a separation of embryonic and crown roots was possible in the described setup, nine plants were grown in the rhizoslides and the whole root system was harvested layer by layer. The number of roots per root type in each of the four layers was recorded.

### Verification of fungi reduction

One consequence of the longer cultivation period was fungal infection. The most prominent fungus was *Chromelosporium fulvum*. To verify that the fungicide treatment could successfully reduce the number of fungal infections without an influence on root morphology, a preliminary experiment was done using a modified pouch setup based on the method described by Hund et al. [10]. Every pouch was supplied with nutrient solution over a wick that was hanging in a single 50 mL tube (Greiner, Frickenhausen, Germany). The tube was filled with sterile nutrient solution containing either 0 g L^-1^, 2.5 g L^-1^ or 5 g L^-1^ Malvin (Syngenta Agro AG, Dielsdorf, Switzerland) (n = 9). Tubes were refilled every 48 h. After ten days, images of the root systems were taken, infection rated and plants were harvested for biomass measurements.

### Image acquisition and pre-processing

For standard imaging, images were taken either with a 21 mega pixel full-frame digital single-lens reflex camera (EOS 5D Mark II, Canon, Tokyo, Japan) equipped with a 50 mm lens (compact macro 50 mm f/2.5, Canon, Tokyo, Japan). The resolution of the images was around 0.13 mm/pixel. The camera was equipped with a circular polarization filter (Hama, Augsburg, Germany) and was placed in 1 m distance parallel to the pouch surface. Two studio flash lights (Walimex pro VC 400, Burgheim, Germany) were used to illuminate the rhizoslides. The lights were positioned at an angle of 30° and a distance of 1 m to the left and right in front of the slide, respectively. The front of the lights were equipped with linear polarization filters (Foto Mayr, Dietzenbach, Germany). For each side of the rhizoslide, two images were taken: one illuminated from the right, the other one illuminated from the left. To trigger these staggered left/right flashes, a microcontroller was built and programmed in Arduino 1.0 (http://arduino.cc/en/) to activate a different flash each time the camera trigger was released. The microcontroller connected the camera and the flashes with a computer and was triggered by CanonEOSUtility Software (V2.1 Canon Inc. 2011) (See Additional file 13). Colour 24 bit RGB images were taken and directly stored on the hard drive by the CanonEOSUtility Software. A backlight was used to evaluate the possibility to measure the embryonic roots, covered by the germination paper, by means of their reduced transmission of light compared to the paper. Roots were backlighted with a continuous spotlight and images were taken from the front (f/4; 1/6 s). In case of infrared images, a monochrome CCD camera (Scorpion SCOR-20SO; Point Grey Research, Vancouver, BC, Canada) equipped with a standard lens (25 mm; Cosmicar/Pentax, The Imaging Source, Bremen, Germany) and an infrared interference filter (940 nm; Edmund Optics, Karlsruhe, Germany) was used. For lighting, a LED panel (880/940 nm) or infrared diode-fields (940 nm) were used. The camera had a resolution of 0.22 mm/pixel and the display detail was approximately 10 × 10 cm.

### Hyperspectral microscopy

To identify the wavelength with the maximal contrast between root and background, a Darkfield transmission optical microscope (CytoViva Hyperspectral Imaging System (HSI), Auburn (AL) USA) was used. The reflection of Steel blue germination paper (Anchor Steel Blue Seed Germination Blotter, Anchor Papers Co, USA), light blue (FP3621), blue (FP3644), and white (FP5703) germination paper from Whatman (GE Healthcare Life Science, Glattbrugg, Switzerland) and Sebio grey (FP3236) (Albet-Hahnemuehle S.L, Dassel, Germany) were recorded in the range of 400 to 1000 nm. Measurements were done using Environment for Visualization software (ENVI 4.8, Exelis Visual Information Solutions, Inc., Boulder, USA) that can extract complete spectral information from single or multiple pixels. The reflection spectrum of a white reflectance standard with spectralon served as reference (WS-1-SL, Ocean Optics, Ostfildern, Germany). Spectra resulted from average values of 13 081 pixels per spectrum. Exposure times were 0.21 s for the spectralon, 0.4 s for Steel blue germination paper, Sebio grey and Whatman blue, 0.3 s for Whatman light blue and 0.25 s for Whatman white.

To correct for differences in exposure time, the intensity of the reflection signal at each wavelength was corrected using the following formula:

$$\mathrm{corrected}\;\mathrm{intensity}=\frac{\mathrm{Intensity}\;\mathrm{value}}{\mathrm{exposure}\;\mathrm{time}\;\left(\mathrm{spectralon}\right)∙\mathrm{exposure}\;\mathrm{time}\;\left(\mathrm{sample}\right)}$$

In the following, the intensity at each individual reflection signal wavelength was normalized by dividing through the reflection intensity of the spectralon.

### Image analysis

**Figure 6:**
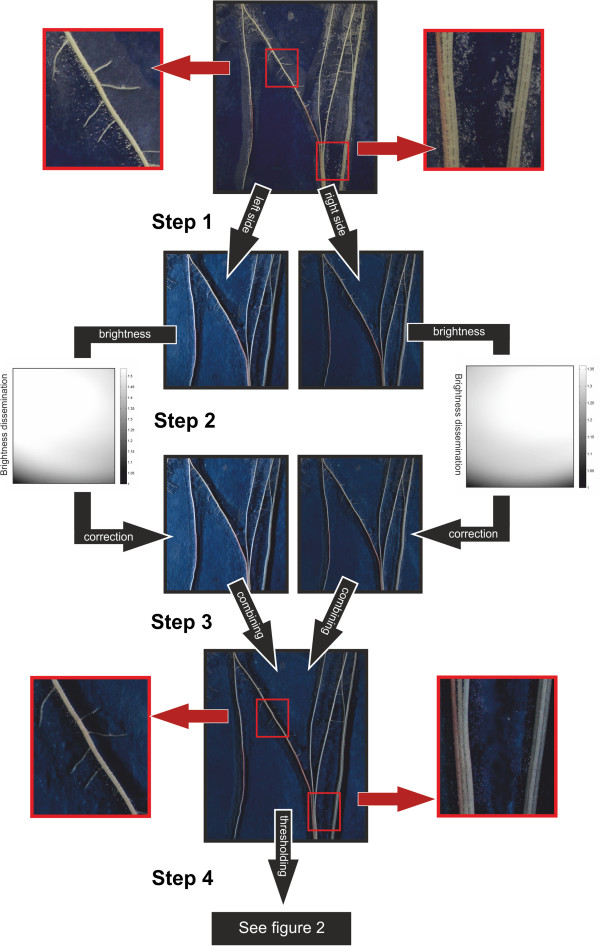
**Work flow of the image processing.** Images in the center row illustrate the workflow and images on right and left the effect of noise reduction. Center row: Step 1: Two images are taken, one with illumination from the right and one with illumination from the left side. Step 2: A correction for inhomogeneous brightness is done. The diagrams illustrate the inhomogeneous brightness for which need to be corrected on the right/left image. Step 3: Images taken with either right or left illumination were combined using minimal tonal value of each pixel. Step 4: A conversion into greyscale using the red channel is done followed by thresholding. For further details see Figure 2. Left and right row: Images show the effect of noise reduction (due to droplets on the inside of the covering foil) (left and right) and an enhanced differentiation between parallel growing roots (right) after the corrections and combination of the two images.

Images, pre-processed in Matlab, were successively analyzed by digital image analysis software developed for root image analysis. The utilized software was WinRhizo (Régent Instruments, Québec, Canada, 2003a) GiARoots or SmartRoot [37,40]. The images were combined to one 24 bit RGB image using Matlab (Version 7.12 The Mathworks, Natick, MA, USA) by keeping only the minimum tonal value present in either image (minimum tonal image). Each of the three RGB colour channels was tested to figure out which channel delivered the best distinction between root and background. A Matlab (Matlab Version 7.12) routine was used to i) balance inhomogeneous illumination, ii) combine the images taken with right or left flash light to one image by using the pixel with the lower tonal value iii) extract the color channel with the highest contrast between roots and background iv) identify each individual by reading the label in the image and v) rename the image with the label content (Figure 6).

Optical differentiation between root and background (called segmentation or thresholding) was done in WinRhizo or GiARoots. In WinRhizo the automated threshold or a manual adaption of the threshold was done by choosing the tonal value with the best noise to root relation. All pixels above this value are assumed as background while all pixels below this value are considered as root. The threshold value of WinRhizo is used for the segmentation of the whole image. In GiARoots an adaptive thresholding or double adaptive thresholding was done. Using the adaptive threshold, the entire image is broken up into smaller square arrays of a certain block size. Within each block, the mean pixel intensity is calculated and all pixels with the same intensity +/- a selectable proportion are considered to be part of the root network, all others are considered to be part of the background. The double adaptive threshold looks at the behavior of the mean intensity as a function of the neighborhood size and classifies the pixel as foreground if a sufficiently large decrease/increase in its values is achieved within a specified range of neighborhood sizes (for details see Galkowskyi et al. 2012).

### Software comparison

To compare the performance of the programs, a dataset of ten root sytems scanned on steel blue germination paper were analysed using WinRhizo, GiARoots or SmartRoot. The images were part of a previous study published in 2009 [10]. In contrast to WinRhizo and GiARoots, Smart root allows for user interference. Therefore, to determine the effect of user interference on root detection by the software, ten images of two contrasting genotypes were measured repeatedly with SmartRoot by three different persons. Furthermore, the time investment performing a topology analysis using WinRhizo or SmartRoot was investigated. The analysis was divided in four steps and time was recorded for every step separately. The classification into steps is not identical for both software packages as their procedures were different, but as close as possible. WinRhizo: 1. Step: Automatic analysis of the image and setting of the segmentation threshold, 2. Step: Excluding non-volitional regions, 3. Step: combining and cutting of root fragments and 4. Step: Allocation of the root order. SmartRoot: 1. Step: Automatic labeling of seminal roots, 2. Step: Manual correction of seminal roots, 3. Step: Automatic labeling of lateral roots and 4. Step: Manual correction of lateral roots.

### Artifical roots

SmartRoot delivers xml files with the position of every node used to analyze the root system. These data, created for every analyzed image, could be used to generate artificial root images by a Matlab script as follows: First points and diameters were read from xml-files written by Smartroot. The points were interpolated by splines to get the complete root line of each single root. Gaussian distributions were positioned along the longitudinal axis of the artificial root in a way that matched full width at half maximum of the distribution with the root diameter at each position. These artificial root systems were used to compare software performance under optimal contrast between root and background.

### Statistics

The variance component of the user interaction experiments were estimated with ASREML-R [74] by setting the factors “user” and “image” as random in a model containing no fixed factor. To estimate the repeatability, we divided the variance of the determined axile and lateral root length within the sampled images (σ^2^_image_) with the overall variance due to image variance plus image-by-user interaction variance (σ^2^_error_).

$$R2=\mathrm{va}r_{\mathrm{image}}/\left(\mathrm{va}r_{\mathrm{image}}+\mathrm{va}r_{\mathrm{error}}\right)$$

The experiment determining the effect of the fungicide on fungal infection and plant growth was a complete randomized block design with 9 replications. Each experimental unit consisted of one rhizoslide containing one plant. A mixed linear model was calculated in ASREML-R as

$$Y_{\mathrm{ij}}=f_{i}+r_{j}+\epsilon_{\mathrm{ij}}$$

where *Y*_*ij*_ is the i^th^ plants in three leaf stage, number of infected plants or plant biomass in the j^th^ replication, f_i_ is the fungicide concentration (i = no fungicide, 2.5 g/L or 5 g/l Captan), *r*_*j*_ is the replication (j = 1, …, 9), and *ϵij* is the residual error. The factor replication was set as random.

## Abbreviations

RSA: Root system architecture; QTL: Quantitative trait loci; NIR: Near-infrared reflectance.

## Competing interests

The authors declare that they have no competing interests.

## Authors’ contributions

The method was conceived by CLM and AH. The development was supported by AW and NK. DM did the software evaluation, analysis and tests. All scripts necessary for imaging and image processing were written by NK. Experiments and data analysis were performed by CLM. Technical equipment was developed and constructed by AH, NK and CLM. All authors read and approved the final manuscript.

## Supplementary Material

Additional file 1 **Separation of embryonic and crown roots based on the paper sandwich method (Figure**1**C and D).** Black bars indicate crown roots growing on top of the paper surface and grey bars indicate crown roots growing under the paper. A replicate represents one rhizoslide planted with one plant. Click here for file

Additional file 2 **Fungicide effect.** Vigor traits measured for plants treated with Captan (2.5 g L^-1^ or 5 g L^-1^) and plants with no fungicide (control). N = 9. Significance level p < 0.001(***); p < 0.01(**); p < 0.05(*); p < 0.1(.). Click here for file

Additional file 3 **Images of Chromelosporium fulvum. A:** Image taken with a consumer camera, **B:** magnifying glass 33 times magnified, **C:** microscope 1000 times magnified. Click here for file

Additional file 4 **Channel separation and NIR lighting. A**: Comparison of the conversion into greyscale images using either the blue, green or red channel or all three channels (grey). **B**: Images taken of roots growing on steel blue, Whatman blue, Whatman white or Sebio grey germination paper using NIR front or backlighting. Click here for file

Additional file 5 **List of programs suitable for 2D root system analysis.** List includes relevant root traits accessible with these programs. Click here for file

Additional file 6 **Thresholding done with four different thresholding routines.** In WinRhizo either an automatic selection or a manual adaptation of the tonal value was chosen. In GiARoots the adaptive threshold and the double adaptive threshold were tested. Red circles indicate the loss of lateral roots due to segmentation and blue circles the difficulties to separate parallel growing roots. Click here for file

Additional file 7 **Correlation of total root length, surface and diameter between WinRhizo, SmartRoot and GiARoots. A**: Based on original images of the roots. **B**: Based on images of the artificial roots. Click here for file

Additional file 8 **Time costs for a topology analysis using WinRhizo or SmartRoot.** The analysis was divided into four steps and after every step the time was recorded. The classification into steps is not identical for both software packages as their procedures were different, but as close as possible. WinRhizo: 1. Step: Automatic analysis of the image and setting of the segmentation threshold, 2. Step: Excluding non-volitional regions, 3. Step: Combining and Cutting of root fragments and 4. Step: Allocation of the root order. SmartRoot: 1. Step: Automatic labeling of seminal roots, 2. Step: Manual correction of seminal roots, 3. Step: Automatic labeling of lateral roots and 4.Step: Manual correction of lateral roots. Data points are mean values (n = 5) ± standard deviation). Click here for file

Additional file 9 **Root traits measured with WinRhizo, SmartRoot and GiARoot listed for one exemplary root.** Root traits were either calculated by the program (direct) or calculated by the user based on the output (indirect). In WinRhizo the length of a single root must be calculated. Therefore, all traits based on single root measurements could not be directly extracted from the output file. All these traits are marked with ”*”. Click here for file

Additional file 10 **Modified output tables of WinRhizo (A) and SmartRoot (B).A**: WinRhizo divides the root system in so called axis and links. A link is a segment on a root 0^th^ order or a 1^st^ order root. An axis is a group of connected links. The yellow highlighted parts are informations about the links and the orange regions about the axis. Green highlighted is the summarizing section. In the second column appears operator (axis, link or summary (DEV-) and in the following columns traits describing this segments are listed. **B**: SmartRoot organizes the data based on roots. In the second column appears the root notation chosen by the user and in the following columns traits describing this root are listed. Values in **A** and **B** are measured for an exemplary root system. Click here for file

Additional file 11 Constructional drawing of the rhizoslides. Click here for file

Additional file 12 **Sandwich method to separate embryonic and crown roots. A**: Granulate substrate to avoid incidence of light. **B**: Potassium polycarbonate on top of the seed to moisten the seed and the mesocotyl. **C**: Crown roots emerging on top of the germination paper edge surrounded by Potassium polycarbonate. Click here for file

Additional file 13 **Diagram of circuit connected to Arduino.** Camera focus and release are controlled by an optocoupler as well as right and left illumination. The illumination is connected to a relay to switch the higher current of the LEDs. Click here for file
