# Supplementary figures and images for: Rhizoslides: paper-based growth system for non-destructive, high throughput phenotyping of root development by means of image analysis
Source: Plant Methods. 2014 May 27;10:13. doi: 10.1186/1746-4811-10-13 (PMC4105838; doi:10.1186/1746-4811-10-13)

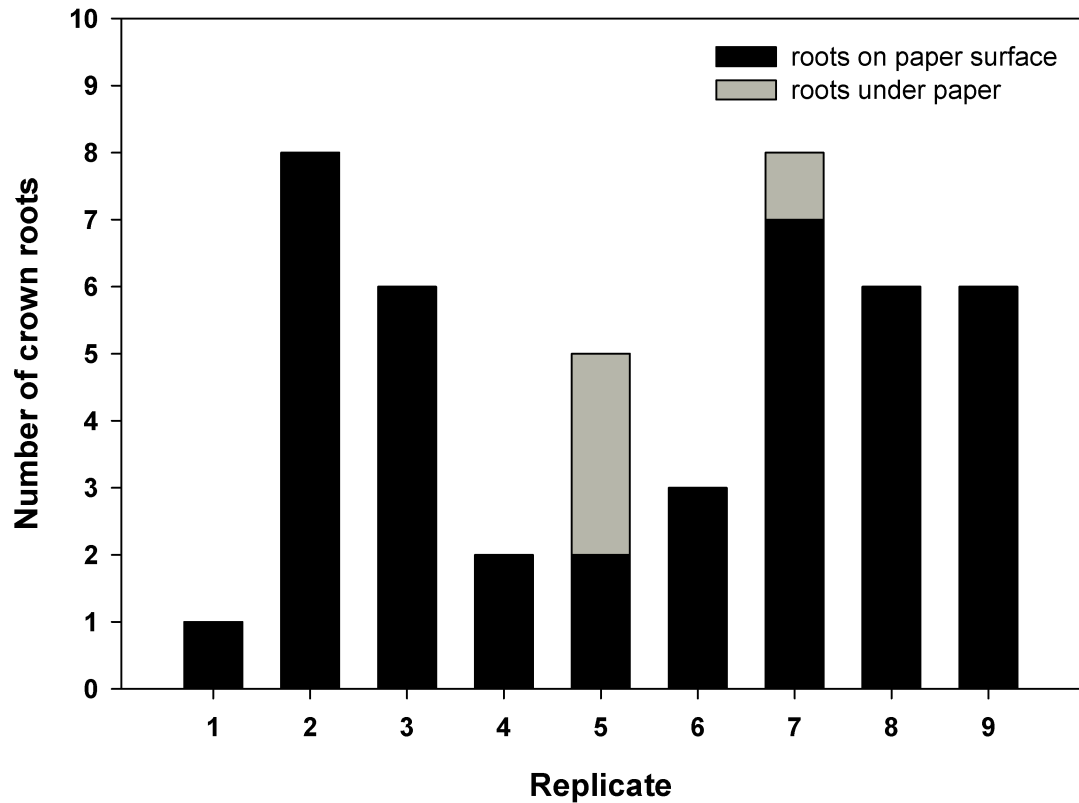

Supplement: Additional file 1 — Separation of embryonic and crown roots based on the paper sandwich method (Figure1C and D). Black bars indicate crown roots growing on top of the paper surface and grey bars indicate crown roots growing under the paper. A replicate represents one rhizoslide planted with one plant. [file 1746-4811-10-13-S1.pdf]

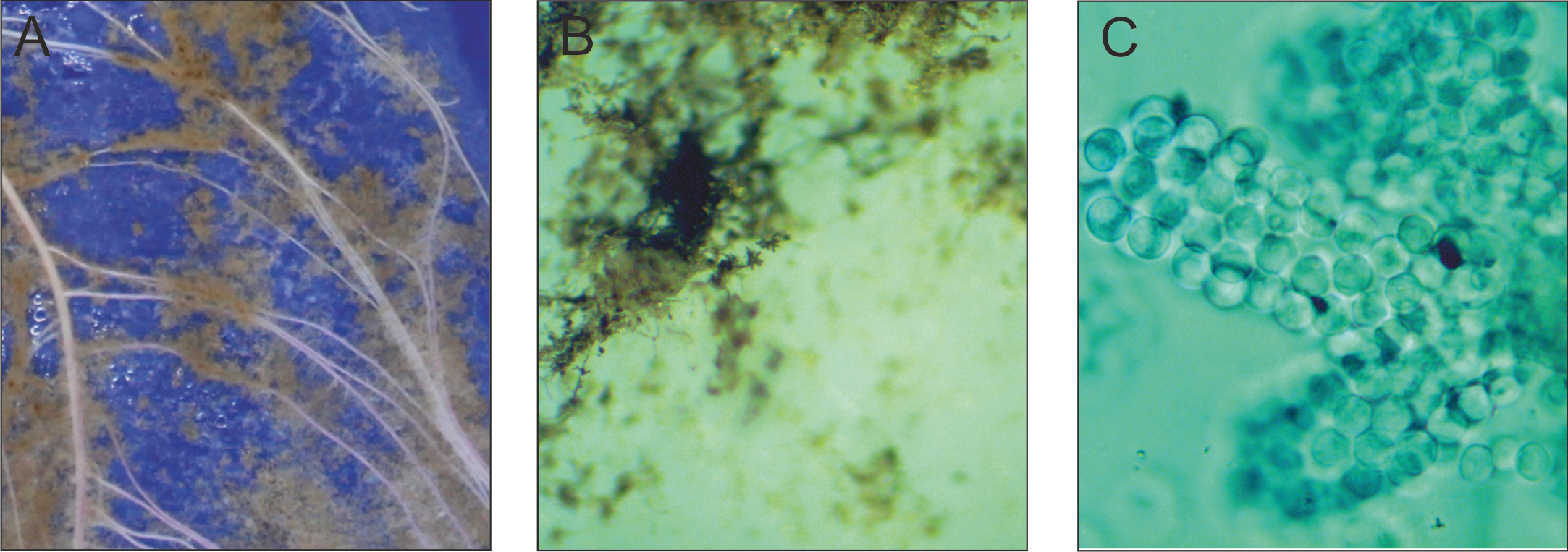

Supplement: Additional file 3 — Images of Chromelosporium fulvum. A: Image taken with a consumer camera, B: magnifying glass 33 times magnified, C: microscope 1000 times magnified. [file 1746-4811-10-13-S3.png]

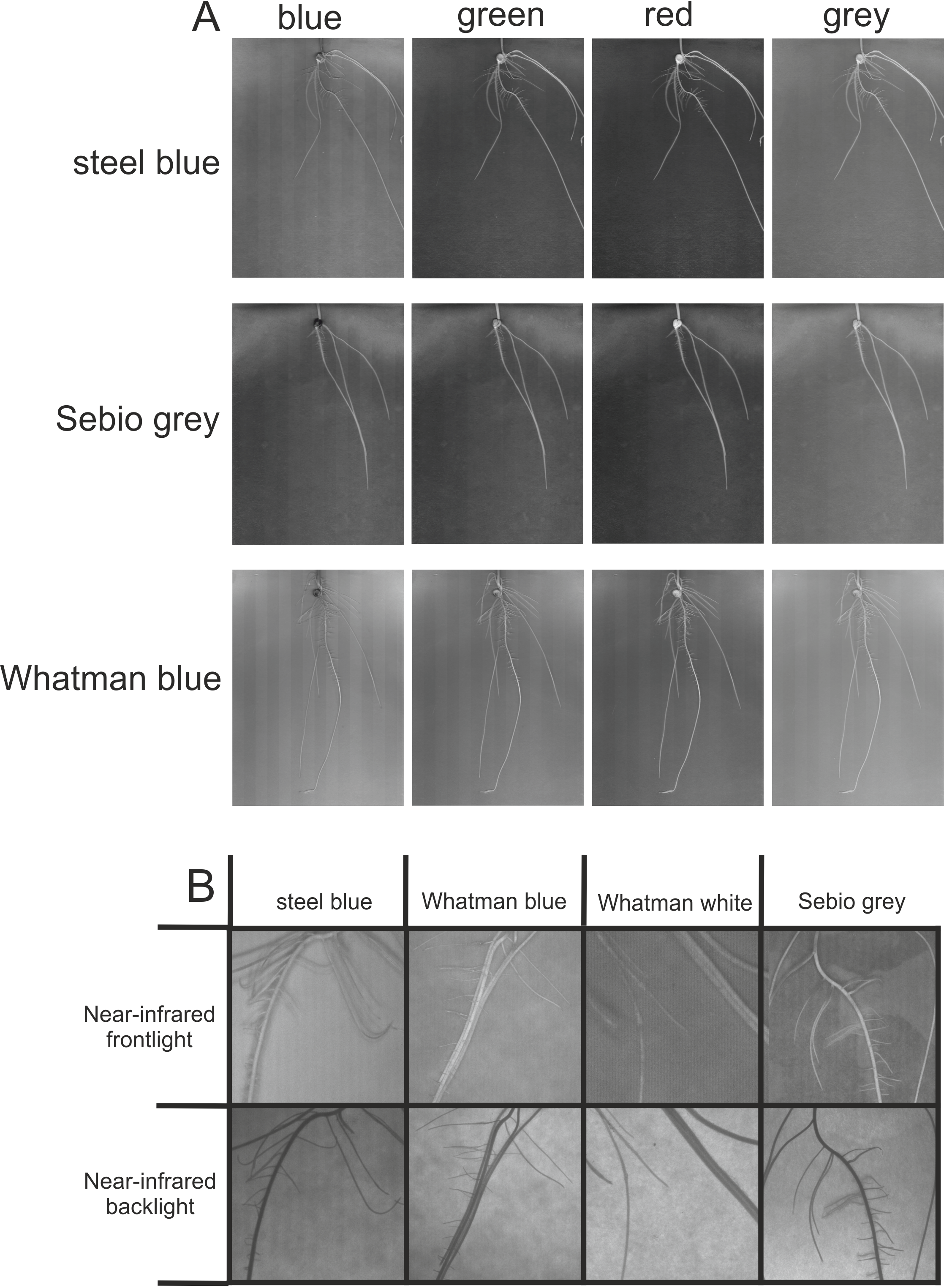

Supplement: Additional file 4 — Channel separation and NIR lighting. A: Comparison of the conversion into greyscale images using either the blue, green or red channel or all three channels (grey). B: Images taken of roots growing on steel blue, Whatman blue, Whatman white or Sebio grey germination paper using NIR front or backlighting. [file 1746-4811-10-13-S4.png]

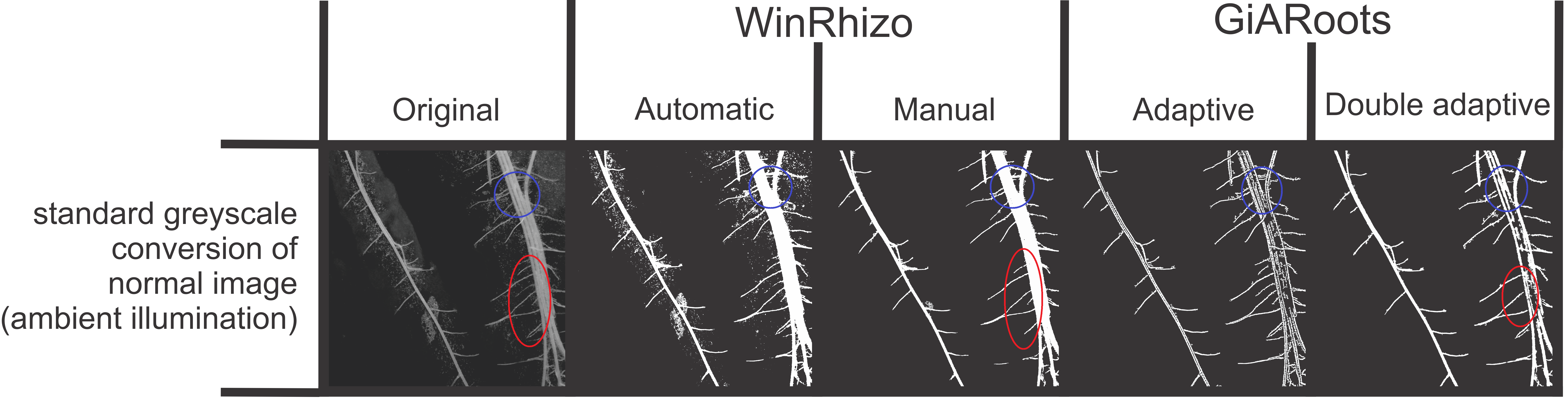

Supplement: Additional file 6 — Thresholding done with four different thresholding routines. In WinRhizo either an automatic selection or a manual adaptation of the tonal value was chosen. In GiARoots the adaptive threshold and the double adaptive threshold were tested. Red circles indicate the loss of lateral roots due to segmentation and blue circles the difficulties to separate parallel growing roots. [file 1746-4811-10-13-S6.png]

**A**

Total root length (cm)

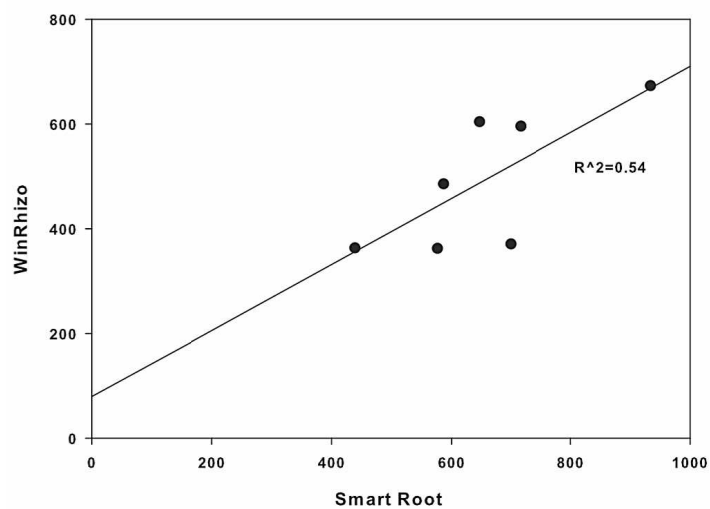

Diameter

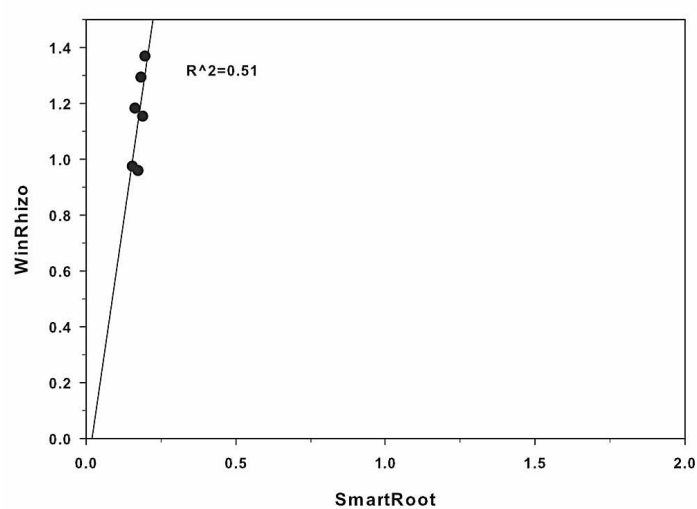

Total root length (cm)

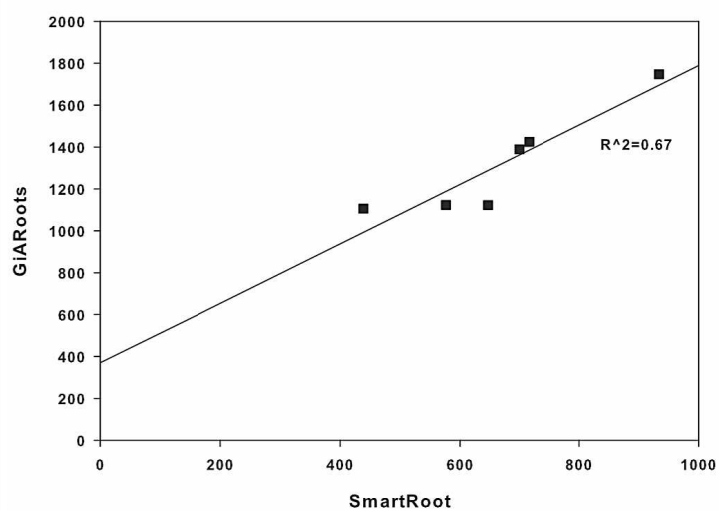

Diameter

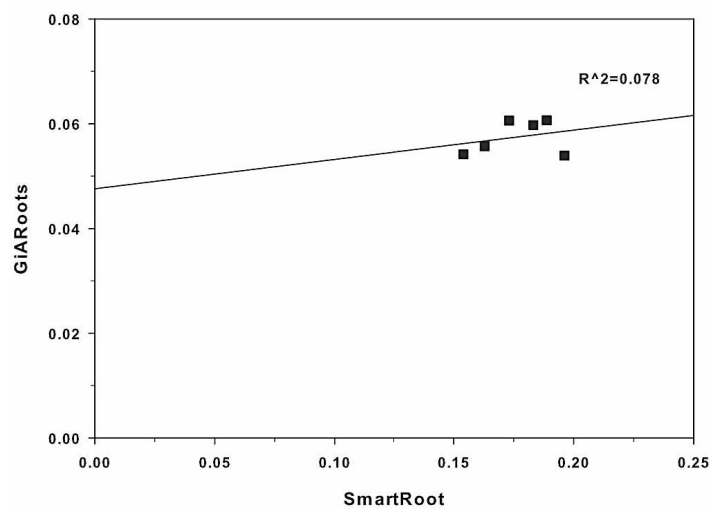

Total root length (cm)

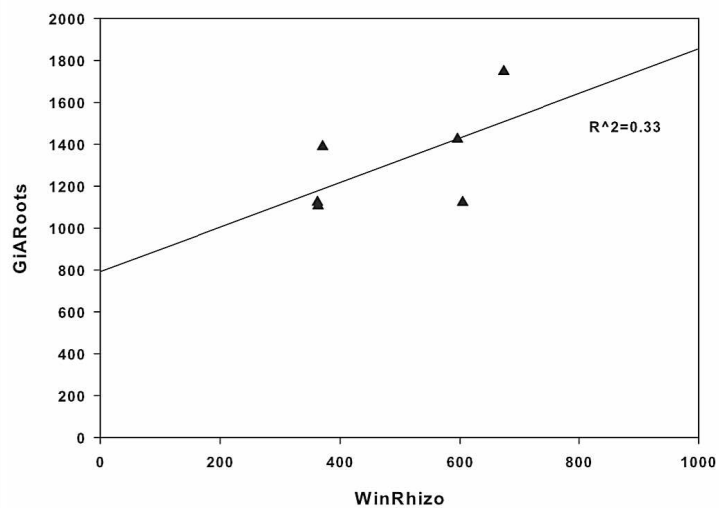

Diameter

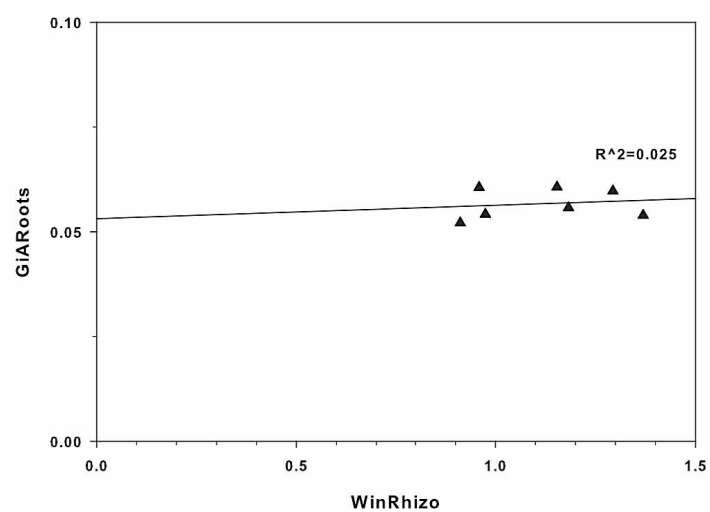

Supplement: Additional file 7 — Correlation of total root length, surface and diameter between WinRhizo, SmartRoot and GiARoots. A: Based on original images of the roots. B: Based on images of the artificial roots. [file 1746-4811-10-13-S7.zip › 1153854852120962_add7A.pdf]

**B**

Total root length (cm)

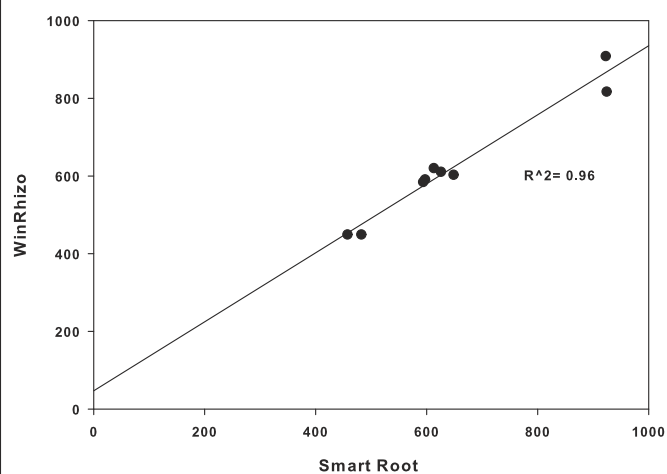

Diameter

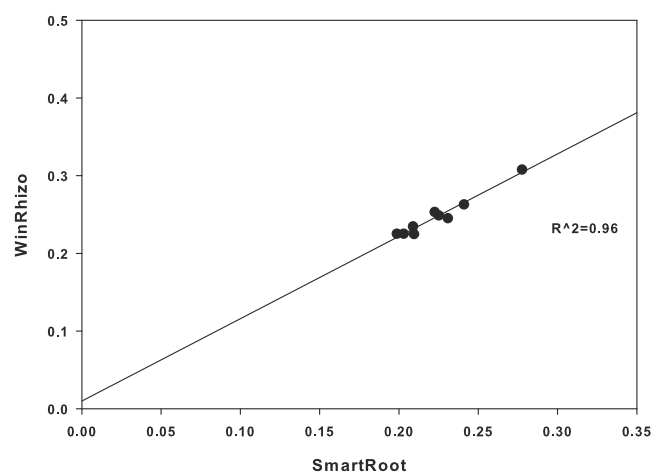

Total root length (cm)

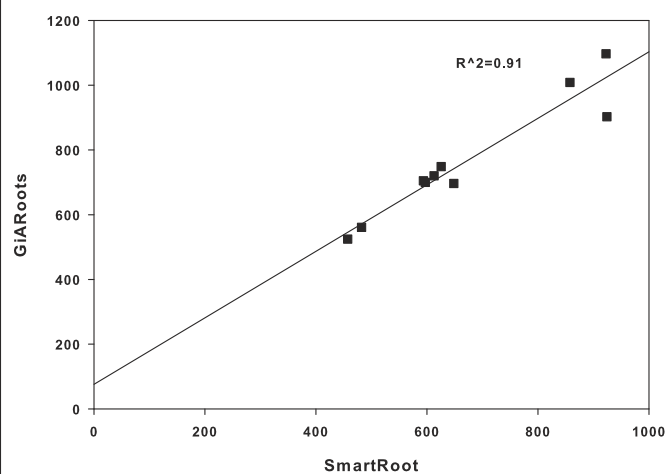

Diameter

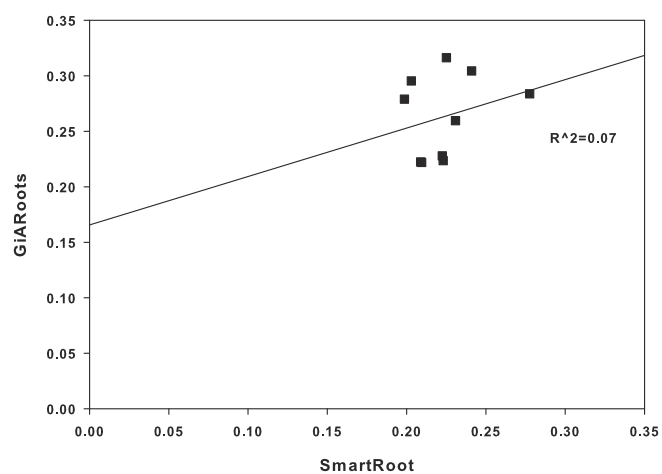

Total root length (cm)

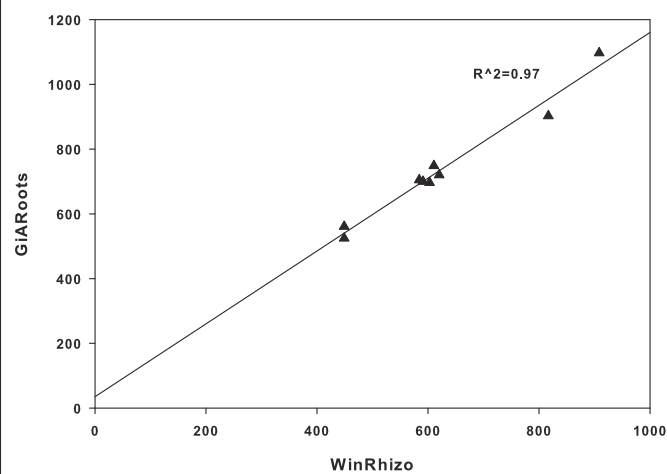

Diameter

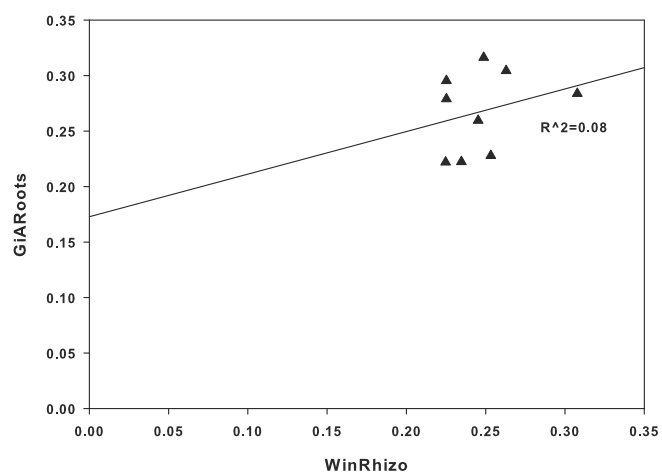

Supplement: Additional file 7 — Correlation of total root length, surface and diameter between WinRhizo, SmartRoot and GiARoots. A: Based on original images of the roots. B: Based on images of the artificial roots. [file 1746-4811-10-13-S7.zip › 1153854852120962_add7B.pdf]

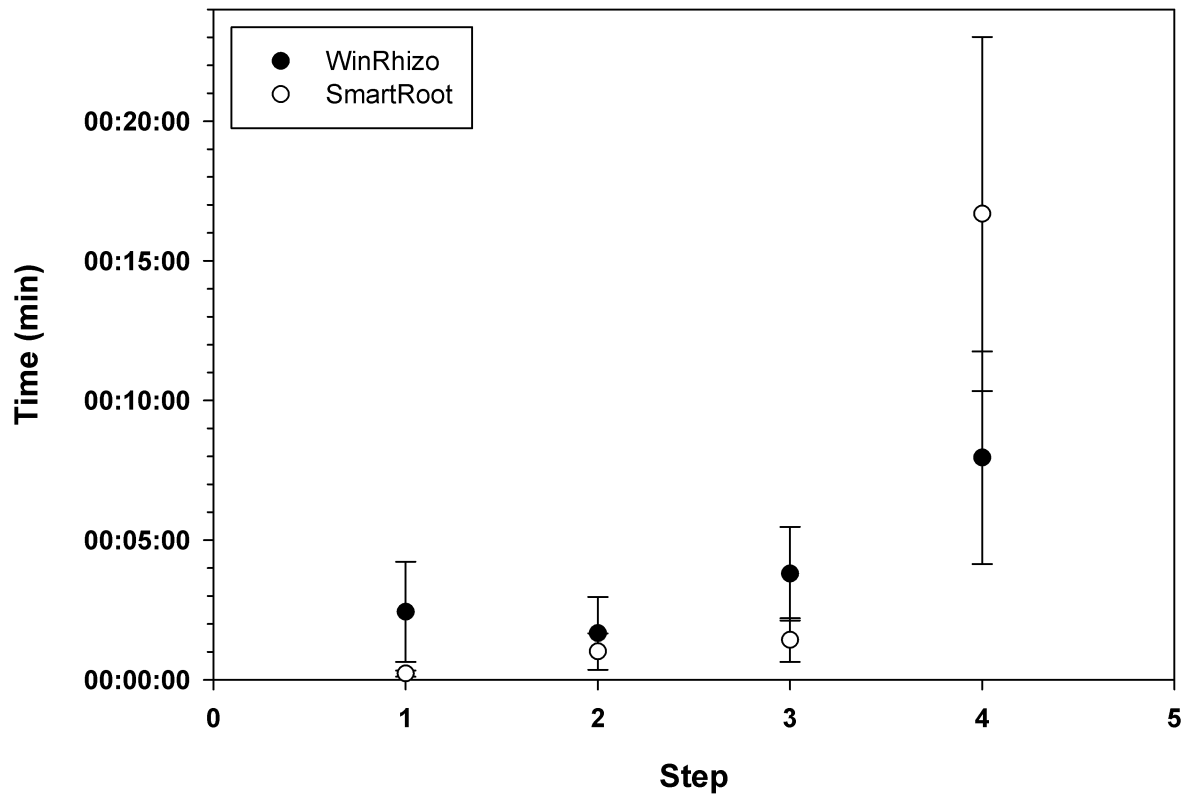

Supplement: Additional file 8 — Time costs for a topology analysis using WinRhizo or SmartRoot. The analysis was divided into four steps and after every step the time was recorded. The classification into steps is not identical for both software packages as their procedures were different, but as close as possible. WinRhizo: 1. Step: Automatic analysis of the image and setting of the segmentation threshold, 2. Step: Excluding non-volitional regions, 3. Step: Combining and Cutting of root fragments and 4. Step: Allocation of the root order. SmartRoot: 1. Step: Automatic labeling of seminal roots, 2. Step: Manual correction of seminal roots, 3. Step: Automatic labeling of lateral roots and 4.Step: Manual correction of lateral roots. Data points are mean values (n = 5) ± standard deviation). [file 1746-4811-10-13-S8.pdf]

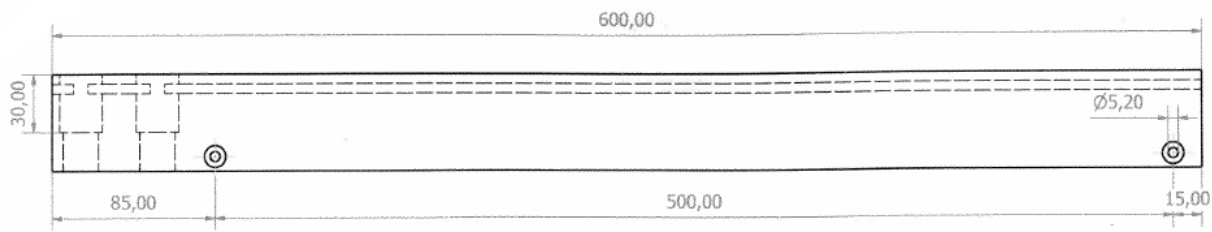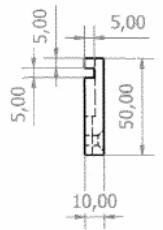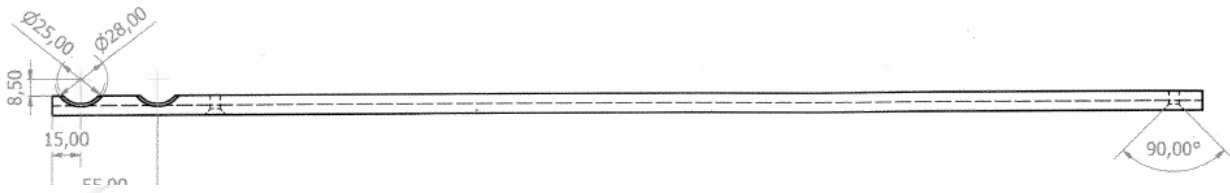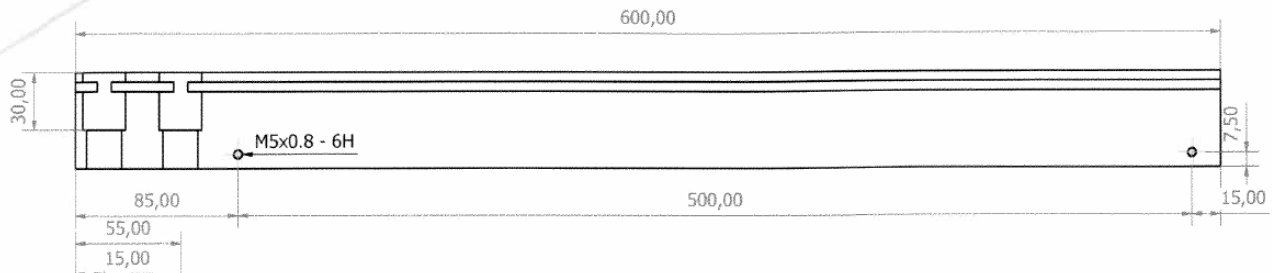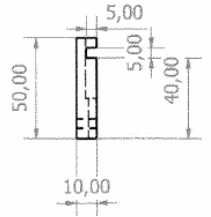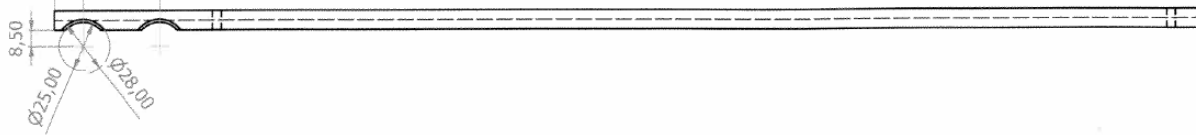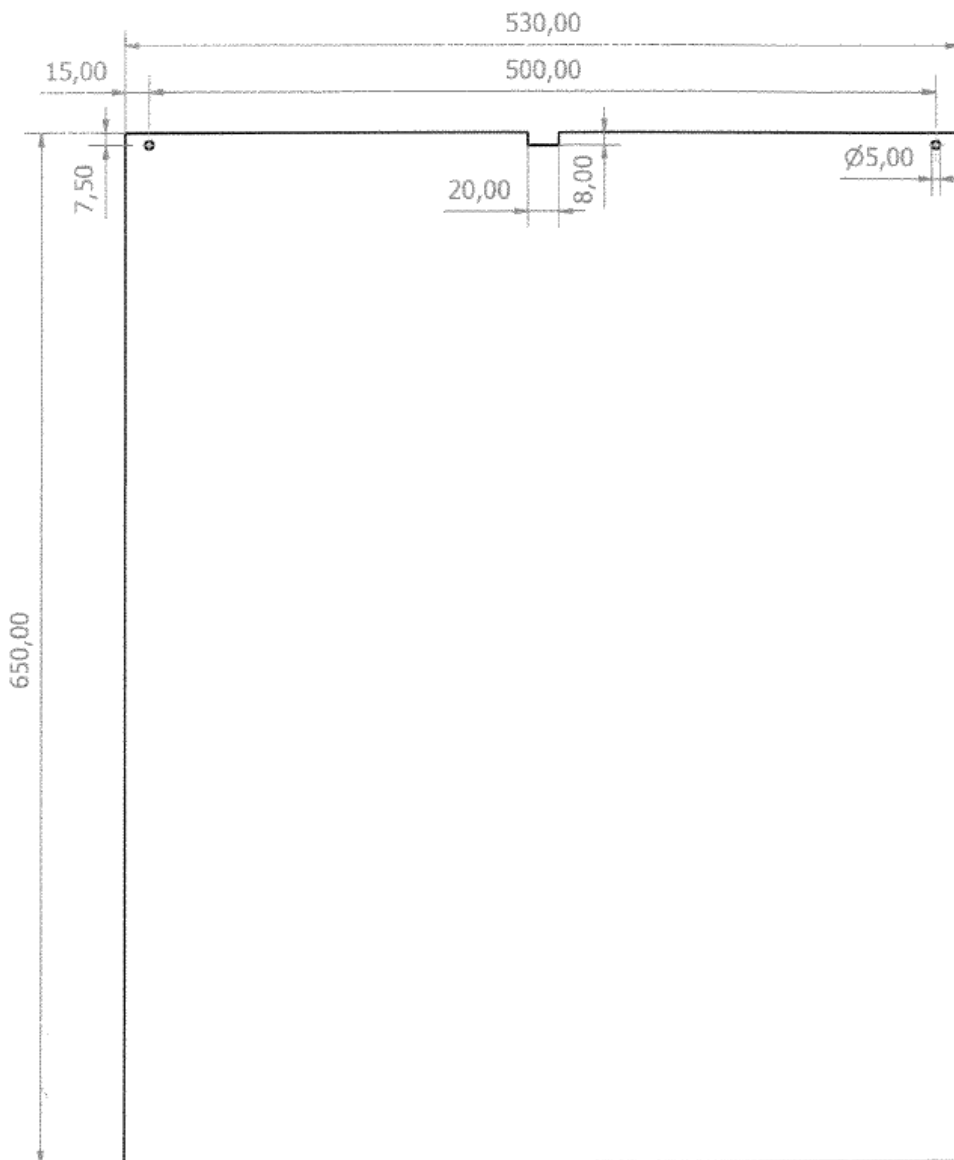

4,00

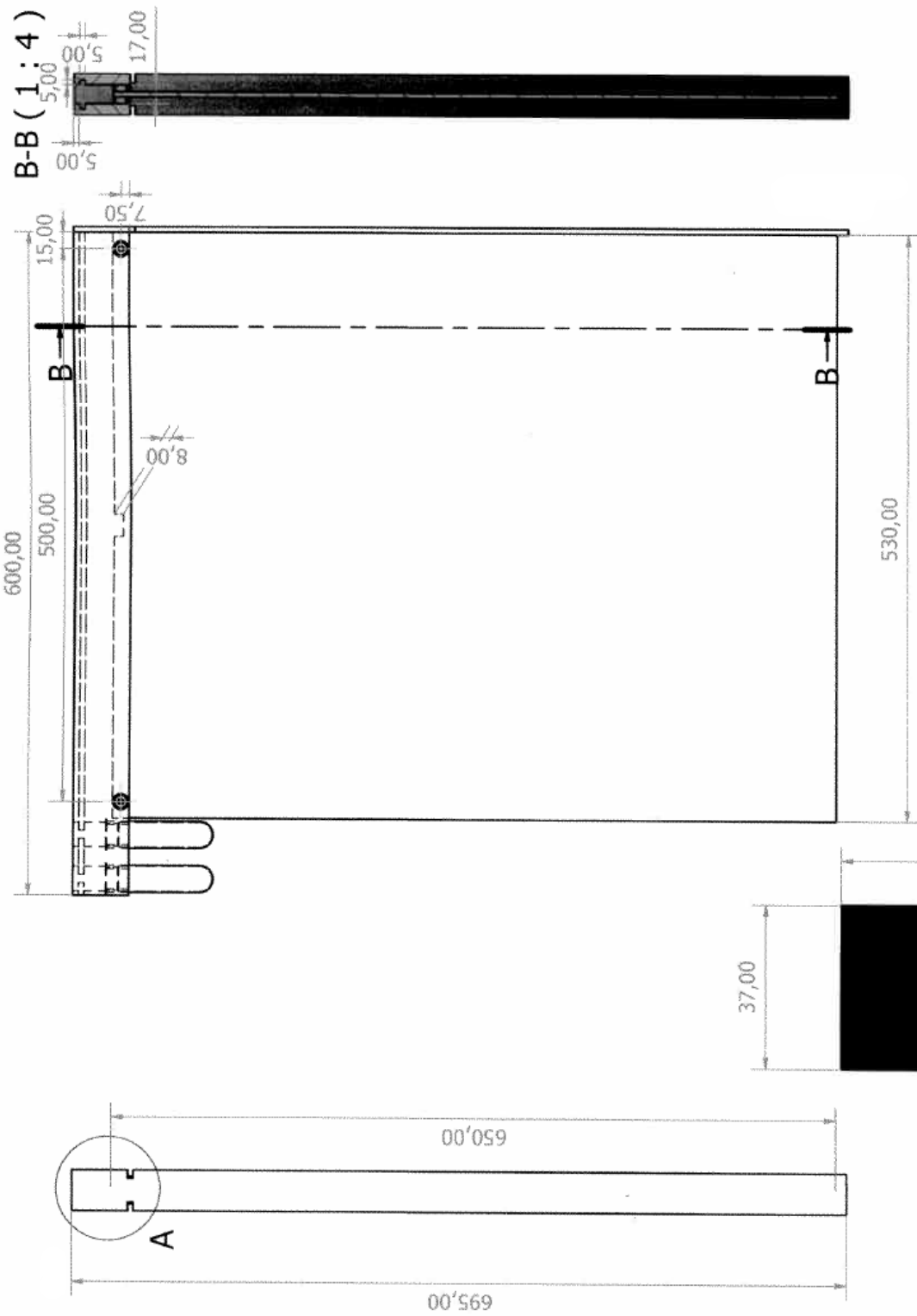

flat washer

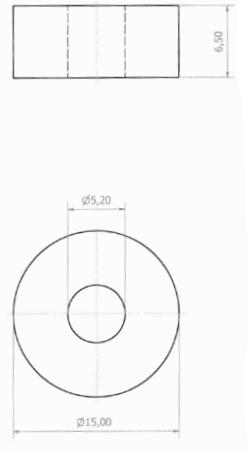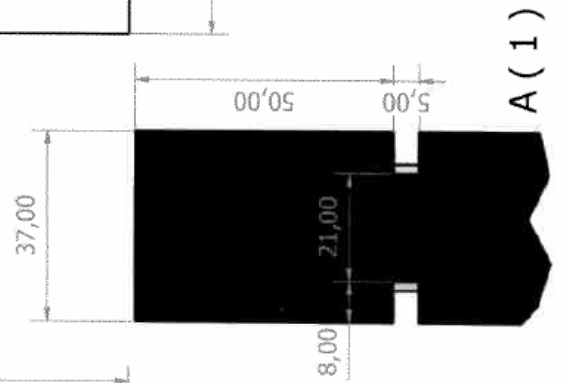

A(1)

Supplement: Additional file 11 — Constructional drawing of the rhizoslides. [file 1746-4811-10-13-S11.pdf]

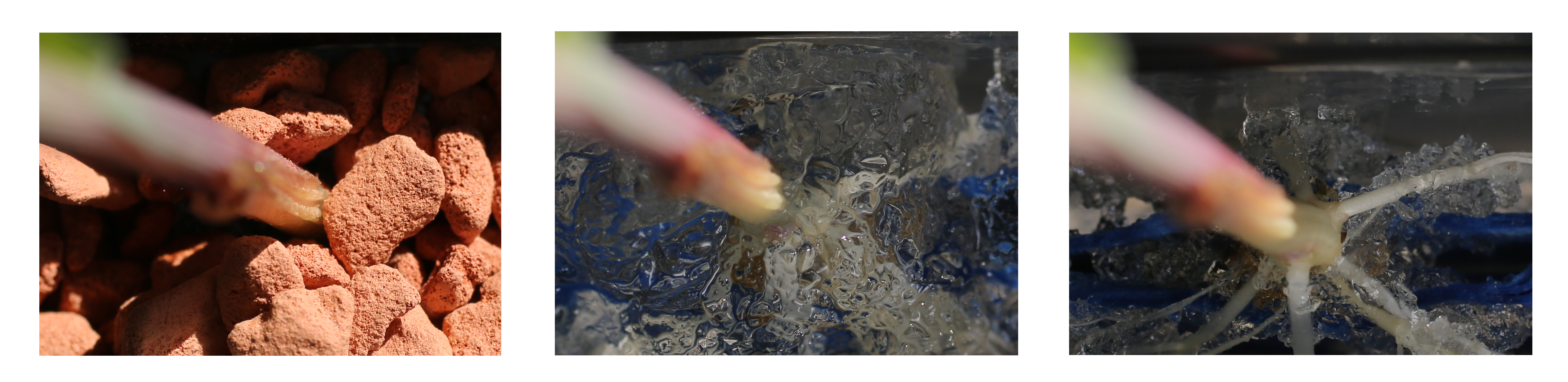

Supplement: Additional file 12 — Sandwich method to separate embryonic and crown roots. A: Granulate substrate to avoid incidence of light. B: Potassium polycarbonate on top of the seed to moisten the seed and the mesocotyl. C: Crown roots emerging on top of the germination paper edge surrounded by Potassium polycarbonate. [file 1746-4811-10-13-S12.png]

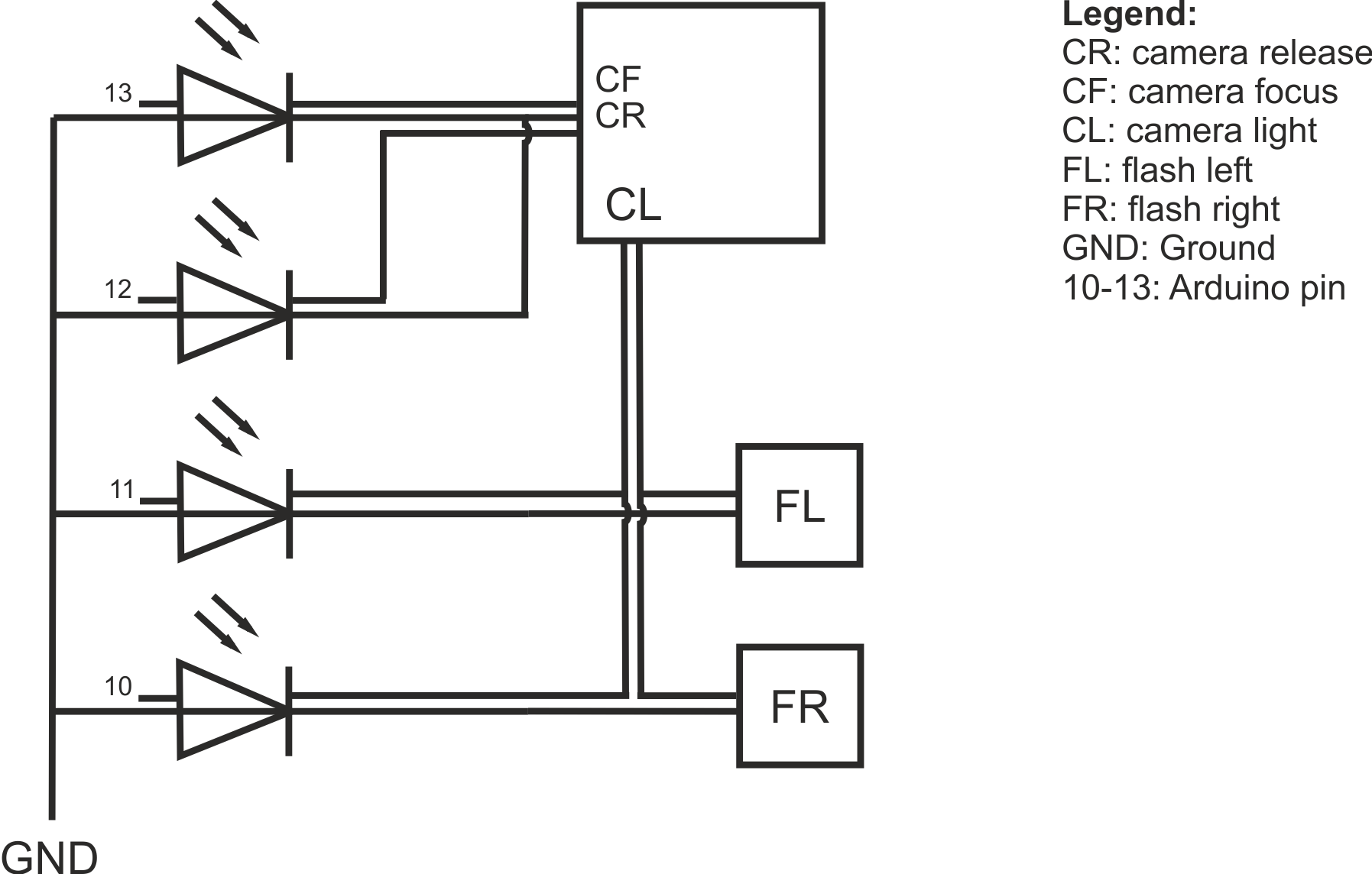

Supplement: Additional file 13 — Diagram of circuit connected to Arduino. Camera focus and release are controlled by an optocoupler as well as right and left illumination. The illumination is connected to a relay to switch the higher current of the LEDs. [file 1746-4811-10-13-S13.png]
