# Supplementary material for: Rhizoslides: paper-based growth system for non-destructive, high throughput phenotyping of root development by means of image analysis
Source: Plant Methods. 2014 May 27;10:13. doi: 10.1186/1746-4811-10-13 (PMC4105838; doi:10.1186/1746-4811-10-13)
Supplement: Additional file 2 — Fungicide effect. Vigor traits measured for plants treated with Captan (2.5 g L-1 or 5 g L-1) and plants with no fungicide (control). N = 9. Significance level p < 0.001(***); p < 0.01(**); p < 0.05(*); p < 0.1(.). [file 1746-4811-10-13-S2.pdf]

Treatment	percentage of plants in 3 leave stage	number of infected plants/total	dry weight (g)	
			mean	STD
**No fungicide (control)**	63	9/9	0.274	0.047
**Malvin 2.5g L^{-1}**	29	1/9	0.254	0.069
**Malvin 5g L^{-1}**	29	1/9	0.282	0.078

ANOVA	percentage of plants in 3 leave stage	Number of infected plants	dry weight
**fungicide**	n.s.	***	n.s.
**concentration**	.	***	n.s.
