# Supplementary material for: Rhizoslides: paper-based growth system for non-destructive, high throughput phenotyping of root development by means of image analysis
Source: Plant Methods. 2014 May 27;10:13. doi: 10.1186/1746-4811-10-13 (PMC4105838; doi:10.1186/1746-4811-10-13)
Supplement: Additional file 5 — List of programs suitable for 2D root system analysis. List includes relevant root traits accessible with these programs. [file 1746-4811-10-13-S5.pdf]

| Analysis | Parameter | SmartRoot | WinRHIZO | EZ - RHIZO | DART | RootReader2D | DigiRoot | RootTrace | GiARoot |
|---|---|---|---|---|---|---|---|---|---|
| | root length | yes | yes | yes | yes | yes | yes | yes | yes |
| **global** | diameter | yes | yes | no | no | no | yes | no | yes |
| | number of roots | yes | no | yes | yes | yes | yes | yes | yes |
| | max. length lateral root | yes | no | yes | yes | yes | yes | no | no |
| | angle | yes | no | yes | yes | yes | yes | no | no |
| | root density | yes | no | yes | yes | no | yes | no | no |
| **detail** | length basal branching zone | yes | no | yes | yes | no | no | no | no |
| | length apical branching zone | yes | no | yes | yes | no | no | no | no |
| | length branching zone | yes | no | yes | yes | no | no | no | no |
| | growth | yes | no | yes | yes | no | no | yes | yes |
| | network depth | no | no | no | no | no | no | no | yes |
| | network width | no | no | no | no | no | no | no | yes |
| | network expansion | no | no | no | no | no | no | no | yes |
| **root types** | primary, seminal, lateral, crown root | yes | partly | yes | yes | yes | n.a. | n.a. | no |
| **Processing** | | semiautomated | (semi-) automated | semiautomated | manual | automated | semiautomated | semiautomated | automated |
| **Source** | | Lobet et al. 2011 | WinRhizo Inc. | Armengaud et al. 2009 | Le Bot et al. 2010 | Clark et al. 2013 | Stefanelli 2009 | Naeem et al. 2011 | Galkowsyki et al. 2012 |
