# Supplementary material for: Rhizoslides: paper-based growth system for non-destructive, high throughput phenotyping of root development by means of image analysis
Source: Plant Methods. 2014 May 27;10:13. doi: 10.1186/1746-4811-10-13 (PMC4105838; doi:10.1186/1746-4811-10-13)
Supplement: Additional file 9 — Root traits measured with WinRhizo, SmartRoot and GiARoot listed for one exemplary root. Root traits were either calculated by the program (direct) or calculated by the user based on the output (indirect). In WinRhizo the length of a single root must be calculated. Therefore, all traits based on single root measurements could not be directly extracted from the output file. All these traits are marked with ”*”. [file 1746-4811-10-13-S9.pdf]

Trait	WinRhizo	Calculation	SmartRoot	Calculation	GiARoots	Calculation
**Total root length (cm)**	620.25	indirect	612.85	indirect	720.11	direct
**Length of all seminal roots (cm)**	142.37	indirect	144.63	indirect	NA	-
**Length of all lateral roots (cm)**	477.88	indirect	468.22	indirect	NA	-
**Specific Root Length**	NA	-	NA	-	22.09	direct
**Maximum Number of Roots**	NA	-	NA	-	4	direct
**Median Number of Roots**	NA	-	NA	-	3	direct
**Total surface area (cm^2)**	402.86	indirect	369.52	indirect	515.45	direct
**Average diameter (cm)**	0.25	indirect	0.22	indirect	0.23	direct
**Volume (cm^3)**	NA	-	22.15	indirect	32.60	direct
**TotalProjArea(cm2)**	128.24	indirect	NA	-	NA	
**Network Bushiness**	NA	-	NA	-	1.33	direct
**Number of Connected components**	NA	-	NA	-	2	direct
**Network Depth**	NA	-	NA	-	105.43	direct
**Network Width**	NA	-	NA	-	26.32	direct
**Network Width to Depth Ratio**	NA	-	NA	-	0.25	direct
**Network Area**	NA	-	NA	-	144.30	direct
**Network Convex Area**	NA	-	NA	-	2245.08	direct
**Network Perimeter**	NA	-	NA	-	1529.82	direct
**Network Length Distribution**	NA	-	NA	-	0.40	direct
**Network Solidity**	NA	-	NA	-	0.06	direct
**Minor Ellipse Axis**	NA	-	NA	-	17.58	direct
**Major Ellipse Axis**	NA	-	NA	-	121.73	direct
**Ellipse Axes Ratio**	NA	-	NA	-	0.14	direct
**for every root:**						
**rootOrder**	yes	indirect*	yes		no	-
**parent**	yes	indirect*	yes	direct	no	-
**sibling**	yes	indirect*	yes	direct	no	-
**number children primary**	no	-	yes	direct	no	-
**child density**	yes	indirect*	yes	indirect	no	-
**first child**	yes	indirect*	yes	direct	no	-
**position first child**	no	-	yes	direct	no	-
**last child**	yes	indirect*	yes	direct	no	-
**position last child**	no	-	yes	direct	no	-

**Insertion angle**	yes	indirect*	yes	direct	no	-
**Length of every single root**	no	indirect*	yes	indirect	no	-
**Traits that result from the single root measurements:**	Average diameter of seminals/laterals		Average diameter of seminals/laterals<br>Number of laterals per seminal root<br>Length of primary root<br>Length of the longest lateral (measurable for each seminal root)<br>Length/position of (non-) braching zones<br>Distances between laterals		-	
