# Supplementary material for: Rhizoslides: paper-based growth system for non-destructive, high throughput phenotyping of root development by means of image analysis
Source: Plant Methods. 2014 May 27;10:13. doi: 10.1186/1746-4811-10-13 (PMC4105838; doi:10.1186/1746-4811-10-13)
Supplement: Additional file 10 — Modified output tables of WinRhizo (A) and SmartRoot (B).A: WinRhizo divides the root system in so called axis and links. A link is a segment on a root 0th order or a 1st order root. An axis is a group of connected links. The yellow highlighted parts are informations about the links and the orange regions about the axis. Green highlighted is the summarizing section. In the second column appears operator (axis, link or summary (DEV-) and in the following columns traits describing this segments are listed. B: SmartRoot organizes the data based on roots. In the second column appears the root notation chosen by the user and in the following columns traits describing this root are listed. Values in A and B are measured for an exemplary root system. [file 1746-4811-10-13-S10.pdf]

**A**

| RHIZO 09 | Operator | Date Time | ImageFileName | ImageAcqDevice AndSoftwareInfo | Analysed RegionArea(cm2) | Analysed RegionWidth(cm) | Analysed RegionHeight(cm) | NExclusions | DevOrder Criteria | ImgType CalibMeth TPU Units PxSizeH PxSizeV [CalFile] | PxClassif |
|---|---|---|---|---|---|---|---|---|---|---|---|
| SampleId | COLOR | Name | OwnerGroup | Specifications/NClass | Def Filt/Bg | Length(cm) | ProjArea(cm2) | SurfArea(cm2) | AvgDiam(mm) | Volume(cm3) | Tips |
| SampleId | LINK | Link# | Seedling# | | Length(cm) | ProjArea(cm2) | SurfArea(cm2) | AvgDiam(mm) | EE-EI-II-IL-BL | Angle(deg) | Magnitude |
| SampleId | DEV | Order# | Seedling# | NofLinks | TotalLength(cm) | TotalProjArea(cm2) | TotSurfArea(cm2) | AvgLength(cm) | AvgProjArea(cm2) | AvgSurfArea(cm2) | AvgDiam(mm) |
| SampleId | AXIS | Order# | Seedling# | Axis# | TotalLength(cm) | TotalProjArea(cm2) | TotSurfArea(cm2) | AvgLength(cm) | AvgProjArea(cm2) | AvgSurfArea(cm2) | AvgDiam(mm) |
| | | Daniela | | | 564.8 | 21.4 | 29.6712 | 1 1 5102 7018 | MAG ANG DIAM | Clr Intr cm 0.004199 0.004228 | GreyThdMan- |
| | LINK | 0 | 0 | | 0.68 | 0.07 | 0.21 | 0.97 | BL | | 19.00 |
| | LINK | 1 | 0 | | 0.06 | 0.01 | 0.03 | 1.77 | II | 18.40 | 18.00 |
| | LINK | 2 | 0 | | 1.32 | 0.05 | 0.17 | 0.41 | EI | 99.88 | 1.00 |
| | LINK | 37 | 0 | | 0.81 | 0.03 | 0.10 | 0.39 | EI | 84.01 | 1.00 |
| | LINK | 38 | 0 | | 1.17 | 0.04 | 0.14 | 0.38 | EE | 83.60 | 1.00 |
| | DEV- | 0 | 0 | 17 | 17.47 | 2.20 | 6.92 | 1.03 | 0.13 | 0.41 | 1.44 |
| | DEV- | 1 | 0 | 23 | 11.36 | 0.41 | 1.29 | 0.49 | 0.02 | 0.06 | 0.45 |
| | AXIS | 0 | 0 | 0 | 1.49 | 0.20 | 0.64 | 0.21 | 0.03 | 0.09 | 1.65 |
| | AXIS | 0 | 0 | 1 | 0.12 | 0.02 | 0.07 | 0.12 | 0.02 | 0.07 | 1.74 |
| | AXIS | 0 | 0 | 2 | 15.86 | 1.98 | 6.22 | 1.76 | 0.22 | 0.69 | 1.25 |
| | AXIS | 1 | 0 | 19 | 1.17 | 0.04 | 0.14 | 1.17 | 0.04 | 0.14 | 0.38 |
| | AXIS | 1 | 0 | 20 | 0.10 | 0.00 | 0.00 | 0.10 | 0.00 | 0.00 | 0.12 |
| | | | | | | | | | | | |

**B**

| Img | Root | Length | Surface | Volume | Diam | rootOrder | path | parent | LPosParent | insertAng | nChild | childDensity | firstChild | LPosFirstChild | lastChild | LPosLastChild |
|---|---|---|---|---|---|---|---|---|---|---|---|---|---|---|---|---|
| 1 | Lat_1 | 0.05 | 0.00 | 0.00 | 0.02 | 1 | Se_2 | Se_2 | 5.35 | 60.56 | 0 | 0 | null | null | null | null |
| 1 | Lat_2 | 0.34 | 0.03 | 0.00 | 0.03 | 1 | Se_2 | Se_2 | 4.62 | 67.47 | 0 | 0 | null | null | null | null |
| 1 | Lat_3 | 0.12 | 0.01 | 0.00 | 0.03 | 1 | Se_2 | Se_2 | 4.12 | 92.38 | 0 | 0 | null | null | null | null |
| 1 | Lat_19 | 0.06 | 0.03 | 0.00 | 0.06 | 1 | Pr | Pr | 4.35 | 40.87 | 0 | 0 | null | null | null | null |
| 1 | Se_1 | 8.23 | 1.56 | 0.02 | 0.06 | 0 | -1 | -1 | 3.61 | 92.78 | 0 | 0 | null | null | null | null |
| 1 | Se_2 | 12.36 | 2.35 | 0.04 | 0.06 | 0 | -1 | -1 | 0 | 0 | 1 | 0.00 | Lat_5 | 2.65 | Lat_5 | 2.65 |
| 1 | Se_3 | 12.37 | 2.37 | 0.04 | 0.06 | 0 | -1 | -1 | 0 | 0 | 4 | 1.46 | Lat_4 | 3.98 | Lat_1 | 5.35 |
| 1 | Se_4 | 7.15 | 1.91 | 0.04 | 0.08 | 0 | -1 | -1 | 0 | 0 | 9 | 1.21 | Lat_14 | 3.04 | Lat_6 | 6.77 |
| 1 | Pr | 10.54 | 2.63 | 0.06 | 0.08 | 0 | -1 | -1 | 0 | 0 | 0 | 0.00 | null | null | null | null |
